# Carbon Quantum Dot-Based Sensors: Photochemical Principles and Multimodal Applications

**DOI:** 10.3390/ma19091810

**Published:** 2026-04-29

**Authors:** Moamen F. Rabea, Edit Csapó, Marek Wojnicki

**Affiliations:** 1 Faculty of Non-Ferrous Metals, AGH University of Krakow, al. A. Mickiewicza 30, 30-059 Krakow, Poland; 2 Department of Chemistry, Faculty of Science, Tanta University, Tanta 31527, Egypt; 3 MTA-SZTE Lendület “Momentum” Noble Metal Nanostructures Research Group, Department of Physical Chemistry and Materials Science, University of Szeged, Rerrich B. Sqr. 1, H-6720 Szeged, Hungary; juhaszne@chem.u-szeged.hu

**Keywords:** carbon quantum dots (CQDs), sensors, pH, metal ions, fluorescence quenching, ratiometric sensing, failure ratio, electrochemiluminescence

## Abstract

Carbon quantum dots (CQDs) are inherently photochemically active nanomaterials, exhibiting excitation-dependent emission, proton-responsive surface states, and modifiable redox properties, enabling various sensing applications across fluorescence, electrochemistry, and electrochemiluminescence (ECL) modalities. This comprehensive review elucidates their methodologies, including PET-driven “turn-off/on” fluorescence, ratiometric pH sensing, electrocatalytic currents, and co-reactant-amplified ECL, achieving low detection limits for metal ions, biomolecules, and environmental analytes. Surface-mediated responsiveness is essential to CQD performance, offering exceptional sensitivity while also conferring inherent cross-reactivity. Meta-analysis was conducted using data extracted from previously published studies on CQDs for the detection property, in which the failure ratio was computed as the number of unsuccessful detections divided by the total number of tests reported in each study. Additionally, critical examination reveals inconsistencies in the limit of detection (LOD) metrics and mechanistic uncertainties, as well as strategies for enhancing selectivity through rational doping and molecular recognition hybrids.

## 1. Introduction

Quantum dots (QDs) are a class of nanoparticles with electrical, physical, and optical characteristics that vary with size and composition. Their maximum size is equal to the corresponding exciton’s Bohr radius [1,2]. Because of the quantum domain effect and size effect, they have an adjustable range of luminous peaks and a high photoluminescence quantum yield (PLQY) [3,4,5].

In 1981, Alexey Onushchenko and Alexey Ekimov created the first QDs made of CuCl [6]. The quantum size effect on the absorption spectra of QDs made from various metal halides and chalcogenides was investigated throughout the following years [7,8,9,10,11,12,13]. CuInS_2_, CuInSe_2_, CuGaS_2_, AgInS_2_, AgInSe_2_, and AgGaS_2_ QDs are I-III-VI type QDs that have recently demonstrated the benefits of broad emission spectrum coverage, good tunability, large Stokes shifts, long fluorescence life, and anti-photobleaching stability [14,15]. However, they are poisonous for commercial uses, require demanding experimental conditions, are expensive, and have poor crystallinity and a low PLQY value. Additionally, they cannot be used directly in an aquatic environment [15,16,17].

**Figure 1:**
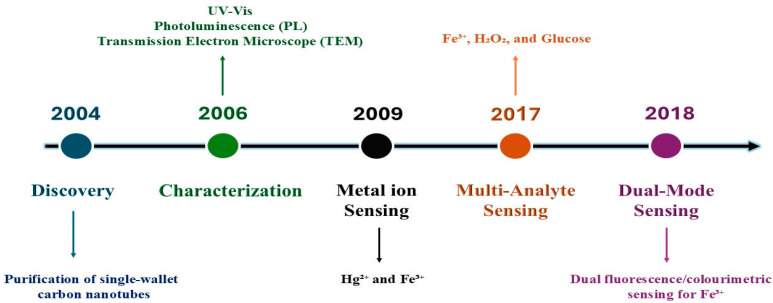
Evolution of CQDs in Sensing.

When Xu et al. purified single-walled carbon nanotubes from arc discharge soot in 2004, they made the initial discovery of CQDs, as depicted in Figure 1 [18]. In 2006, Sun et al. employed carbon quantum dots for the first time when they created CQDs by laser ablation of a carbon target with argon acting as a carrier gas and water vapor present [19]. Since then, numerous scientists have begun to investigate CQDs on a constant basis. They have done so by using a wide range of carbon source precursors (including small molecule organic compounds, carbon fiber materials, biomass-based materials, etc.) to create CQDs using pyrolytic carbonization, hydrothermal, microwave, electrochemical oxidation, and combustion methods [20].

CQDs are zero-dimensional, mono-dispersed, quasi-spherical, and crystalline structures featuring amorphous surface nanoparticles (usually <10 nm) that exhibit remarkable photoluminescence, cost-effectiveness, water solubility, surface tunability, chemical stability, biocompatibility, and low toxicity. In photoexcited states, they both donate and capture electrons [21,22,23]. They have a strong fluorescence intensity even at extremely low concentrations, tunable photoluminescence, high quantum yield, and resistance to photo-bleaching. Because of their inexpensive and eco-friendly synthesis, they provide a sustainable substitute for conventional metal-based QDs [24,25,26].

The advantages they offer have prompted a great deal of study of their usage in a variety of fields, including bioimaging, catalysis, energy-related applications, and especially chemical sensing, which is one of the fields with the fastest rate of technological advancement [27].

Because CQDs may interact with molecules at the interface, their use in sensing has shown great promise. CQDs are versatile and effective at detecting heavy metals because of their high selectivity and sensitivity [28,29]. The ability of pH-responsive CQD-based sensors to convert momentary changes in proton concentration into readily measurable optical signals has drawn considerable attention among the various sensing platforms developed. The development of CQD-based pH sensors toward commercial feasibility and practical use is still hindered, despite remarkable laboratory demonstrations. Real-world applications are frequently hampered by issues related to selectivity, analytical reproducibility, LOD, LOQ, and environmental stability. Furthermore, matrix effects, interfering ions, and batch-to-batch variability in CQD synthesis complicate their analytical reliability.

This review aims to carefully investigate CQD-based sensing platforms, with a special focus on photochemical, electrochemical, and fluorescence-driven mechanisms. The fundamental interactions regulating signal generation, the influence of surface functionalization and heteroatom doping, and the relationship between structural design and analytical performance are all depicted. Furthermore, we critically assess selectivity, sensitivity, LOD/LOQ determination, response time, and practical constraints that now define the gap between academic research and real-world applications. Possible reasons for many metal ions that do not interfere with CQDs are discussed. It aims to offer a cogent framework to direct the logical design of next-generation CQD-based sensors by fusing mechanistic insights with performance measurements and technological factors.

## 2. Fundamental Properties of CQDs Relevant to Sensing

### 2.1. Optical and Photophysical Properties

CQDs exhibit remarkable physical and chemical features attributable to their distinctive chemical structure, with the most notable being their optical capabilities, which encompass photoluminescence, UV-visible absorption characteristics, and fluorescence characteristics and stability. It has been suggested that the PL emission processes of CQDs are caused by surface defects and conjugated π-domains [30]. The process known as photoluminescence (PL) occurs when an external light source irradiates a material, stimulating it to cause light emission. PL offers a comprehensive understanding of the optical properties of CQDs, which are dependent on their surface states and may be influenced by PL mechanisms such as band gap, size, pH, starting material, chemical solvent/environment, and surface functionalization or passivation. Excitation and emission are the two processes that often characterize the photoluminescence behavior of CQDs. It is important to remember that both processes start with a leap between various energy levels. An excited electron returning to its ground state causes PL by recombining with a hole and releasing electromagnetic energy in a narrow, symmetrical energy band that extends from the ultraviolet to the near-infrared spectrum [31]. In general, PL emission occurs at a wavelength that is longer than the excitation wavelength (Stokes PL emission) [32]. However, due to two-photon excitation and anti-Stokes PL emission, CQDs may emit an up-conversion photoluminescence (UCPL) when their wavelength is shorter than the excitation wavelength [33].

CQDs typically exhibit significant light absorption in the ultraviolet and weak light absorption intensity in the near-infrared region. A portion of CQDs also exhibits strong light absorption peaks in the visible spectrum [34,35,36]. The sp^2^ hybridization of a C=C bond π-π* transition from the graphitic cores is represented by one or more distinct absorption peaks in the UV region, typically between 220 and 280 nm. The n-π* transition of C=O, C-N, or C-S and C-O resulting from surface defects is assigned to the 280–350 nm range [37,38]. It should be mentioned that, in addition to the doping of heteroatoms like N and S on the surfaces of CQDs, other factors that may affect the UV absorption spectrum of CQDs include the synthesis technique, raw precursor, pH of the medium, solvent, and alteration of the surface functional groups [38,39]. The addition of different functional moieties, such as amines, carbonyl, hydroxyl, and carboxyl groups, to the surface of CQDs is also crucial because it creates surface defects that serve as energy traps, improving the particles’ photo-reversibility, stability, non-toxicity, biocompatibility, and sensing capabilities [40].

In contrast to classic fluorescent dyes, which often drop quickly as light levels rise, CQDs exhibit good fluorescence stability, a fluorescence intensity that scarcely diminishes under continuous UV irradiation, and a very high resistance to photobleaching [36].

### 2.2. Surface Chemistry and Proton-Responsive Functional Groups

In unmodified CQDs, fluorescence is caused via direct transitions between the core Highest Occupied Molecular Orbital (HOMO) and Lowest Unoccupied Molecular Orbital (LUMO) levels [41]. Passivation of the carbonaceous core by surrounding functional groups, such as -COOH, -OH, -NH_2_, or doping with heteroatoms, significantly influences the optical characteristics of CQDs [42,43,44]. When π-conjugated systems are present along with a small quantity of surface functions, the optical response becomes more prominent. In such instances, the π-conjugated domains play a significant role, with their band structures functioning as the principal emission centers of the carbon-based core, possibly the π to π* transition. This will obviously result in excitation independence due to the single transition mode with a particular energy [45,46]. Surface passivation generates a protective coating on the surface of CQDs, shielding them from impurity adhesion and adding stability. Adding surface-passivating compounds to CQDs increases their quantum yields and fluorescence, making them very optically active. Covalent interactions between functionalizing agents and CQDs can provide materials with outstanding photo reversibility, low toxicity, high stability, and strong biocompatibility [47]. Alcohols, amines, and thiols enhance photoluminescence by reducing surface defects and modifying trap states. They improve emission by reducing non-radiative recombination, increasing solubility, and decreasing cytotoxicity [32,48].

Doping CQDs with heteroatoms, such as N, P, B, and S elements next to carbon in the periodic table, effectively tailors their electronic structure, providing tunable emission, increased quantum yield, and an excitation-independent fluorescence for varied applications [49]. Nitrogen doping improves fluorescence by incorporating functional groups and polyaromatic structures. This modifies the electronic configuration, allowing for tunable emission across the visible spectrum, improved photostability, and increased sensitivity in detecting ions [50,51].

Sulfur doping improves the quantum yield and fluorescence of CQDs by producing defect states that capture excitons and reduce non-radiative losses [52]. Because of its various oxidation states, sulfur can finely control the band gap of CQDs, improving their ability to absorb and emit photons efficiently [53]. Furthermore, sulfur-containing functional groups enhance surface passivation, boost solubility, and provide selective binding capabilities [54].

Phosphorus doping is an effective method for fine-tuning the fluorescence features of CQDs by producing localized energy levels within the band gap, which encourage radiative recombination and allow emission at longer wavelengths [55]. As a pentavalent element, phosphorus provides additional electrons to the carbon structure, modifying its electrical configuration and increasing its luminous efficiency [56]. Furthermore, phosphorus-based functional groups improve the biocompatibility, solubility, and responsiveness to certain analytes, making phosphorus-doped CQDs appropriate for sensing and imaging applications [57].

Because boron and carbon are next to each other in the periodic table of elements and have comparable atomic radius, they share structural and physicochemical characteristics. As a result, doping boron can improve CQD flaws and optical qualities [58]. Doping boron atoms onto CQDs causes a dispersion of positive charges around the carbon atoms because boron atoms are less electronegative than carbon, in contrast to doped elements like N and S [59]. Boron doping can significantly increase the fluorescence intensity or quantum yield (QY) [60].

Due to its biocompatibility, silicon is a good candidate for CQD doping. The Si-CQDs demonstrated favorable water solubility, high photostability, solid-state fluorescence, film-forming ability, and resistance to temperature, alkali, acid, and ionic strength [61].

Since dual-atom-doped CQDs are great prospects for sensitive detection and other applications, several researchers have sought to create them. When nitrogen and sulfur are co-doped into CQDs, their energy level and structure are altered, their fluorescence intensity is increased, and their emission peak position and tunable wavelength are changed [62,63]. J. Yu et al. [64] created N, S CQDs using citric acid and thiourea, which showed exceptional unique surface properties and high quantum yield for the detection of Fe^3+^ ions, displaying extremely sensitive detection limits.

Furthermore, a number of investigations show that surface states that are influenced by protonation–deprotonation equilibria greatly affect CQD fluorescence. pH variations affect charge-transfer kinetics, surface trap density, and on-radiative recombination rates. The interfacial chemistry and emissive behavior of CQDs are crucially regulated by protonation–deprotonation equilibria of surface carboxyl, hydroxyl, and amino groups. While protonation of -NH_2_ groups modifies electron-donating capabilities and photoinduced electron transfer pathways, deprotonation of -COOH groups increases electrostatic attraction and coordination with hard Lewis acids like Fe^3+^. As a result, changes in surface charge density and trap-state populations cause CQD fluorescence intensity and emission profiles to strongly vary on pH [65,66].

It is commonly acknowledged that the primary sites of photoluminescence and chemical sensitivity are surface states, which are produced by functional groups that contain oxygen, nitrogen, and heteroatoms. Trap states that localize excitons and control emission energy levels can be introduced by surface oxidation, defects, and functional groups; these effects frequently outweigh the contribution of quantum confinement effects. Interactions with analytes like metal ions directly alter trap levels in these surface states, which function as sensing-active centers. This modulation of radiative and non-radiative recombination pathways produces detectable variations in fluorescence intensity or wavelength [46,49,55,56].

### 2.3. Charge Transfer and Redox Sensitivity

CQDs’ sensing function is largely dependent on charge-transfer mechanisms. When photoexcited, electrons in surface emissive states can go to analytes that lack electrons, such as Fe^3+^, which causes photoinduced electron transfer to quench fluorescence. Furthermore, static charge-transfer routes that inhibit radiative recombination can be introduced by ground-state complex formation between metal ions and surface functional groups. CQD sensitivity to redox-active species is further increased by the intrinsic redox activity of surface quinone-like and heteroatom-doped sites. All of these findings show that surface-mediated charge-transfer and redox interactions are essentially responsible for CQD fluorescence sensing [67,68]. For instance, CQDs can be utilized as a sensor for both iron (II) and iron (III) induced significant quenching. Because iron (III) is common in fly ash and wastewater, sensing metrics like sensitivity, LOD, and Stern-Volmer constant were benchmarked using it [21,57,69]. The fluorescent probes exhibit a greater quenching impact, lower LOD, and higher sensitivity for Fe^3+^ than for Fe^2+^ ions. This may be related to the higher number of empty orbitals in Fe^3+^ (Hard Lewis Acid), which affects the charge density, ionic radius, and quenching potential to interact with the surface hydroxyl/carboxyl groups (Hard Lewis Bases). When compared to less charged, borderline ions like Fe^2+^, hard–hard interactions are thermodynamically more favorable and result in a greater binding affinity between Fe^3+^ and oxygen functional groups on CQDs [70,71].

## 3. Photochemical CQD-Based Sensors

### 3.1. Fluorescence “Turn-Off” Sensors

One of the most often reported CQD-based sensing techniques is the use of fluorescence “turn-off” sensors. When CQDs interact with analytes in these systems, photophysical or photochemical reactions cause the fluorescence intensity to diminish [72].

Both static (SQE) and dynamic quenching (DQE) can result in the quenching of fluorescence [72]. Energy transfer includes any of the following types: Förster resonance energy transfer (FRET), Dexter energy transfer (DET), surface energy transfer (SET), an inner filter effect (IFE), or photoinduced electron transfer (PET) [68,73].

SQE is caused by a strong contact between the quenchers and the fluorescent probe, which forms non-fluorescent complexes in the ground state and causes the fluorescence intensity to be quenched. However, the emergence of new absorption peaks in the UV–visible absorption spectrum indicates that the fluorescence lifetime of CQDs is unaltered. As the temperature rises, the ground-state complex’s stability may decrease, lessening the impact of static quenching [74,75]. On the other hand, excited-state electrons in CQDs collide with metal ions during DQE, resulting in non-radiative transitions that dramatically limit their fluorescence lifetime while leaving the UV absorption spectrum unchanged. The effect of dynamic quenching may intensify as the temperature rises [75]. One particular example of resonance energy transfer, which is controlled by dipole–dipole interactions, is FRET [76]. Since its initial description in 1950, FRET’s great sensitivity and quick response have made it a popular method for optical sensing [76]. In FRET, a second fluorophore (the “acceptor”) receives photonic energy (non-radiative energy transfer) from a first fluorophore (the donor) and subsequently emits it. The Forster distance, which is roughly 10 nm, separates them both [77]. A match between the donor and acceptor’s redox potentials is necessary for DET, an effect that relies on electron transfer rather than photon transfer.

Theoretically predicted by R. Chance et al. in 1978, SET is most frequently observed with (metal) nanoparticles and involves a molecular (organic) dipole and a metallic surface, like that of gold NPs [78]. The inner filter effect (IFE) results from the direct interaction with the fluorophore when the absorption spectrum of the “quencher” in the detection system overlaps the excitation or emission spectra of CQDs [79]. This impact results in a decrease in intensity without molecular interaction, unlike static (ground-state complexation) or dynamic (collisional) quenching. IFE happens when the analyte absorbs at either the excitation wavelength (primary IFE) or the emission wavelength (secondary IFE), merely filtering out a particle’s emission. This occurs when the radiated and re-absorbed lengths are more than 10 nm. Additionally, IFE shows that there is no change in the fluorescence lifetime of CQDs because no new substance is formed [73]. False-positive interpretations of sensing processes could result from failing to consider IFE, especially in systems with strongly absorbing metal ions. Therefore, it is essential to distinguish IFE from static and dynamic quenching processes. Fluorescence lifetime measurements act as a unique feature: both IFE and static quenching maintain the excited-state lifetime, whereas dynamic quenching reduces it. Stern-Volmer analysis (plotting I_0_/I against [Q]) elucidates mechanistic characteristics: linear plots signify actual quenching, while upward curving indicates intersystem crossing effects resulting from increased light reabsorption at higher concentrations. Dilution to low absorbance values confirms IFE if quenching is reversible, as illustrated by houttuynia cordata-derived CQDs for metronidazole detection, where non-linear plots and dilution response validated IFE’s predominance over complexation [80]. Furthermore, it is advisable to conduct a quantitative assessment of the spectral overlap between the UV–Vis absorption of the analyte and the excitation/emission spectra of CQDs, rather than relying on visual assessment [81,82].

PET can be explained by the electron transfer that took place between the CQDs and the quencher, creating the cation radical and the anion radical, respectively. This procedure formed a complex between the electron donor and the electron receptor that can return to the ground state without releasing a photon. Both oxidative and reductive PET were present. Reductive PET involved CQDs receiving electrons from the electron donor as an electron receptor. The opposite of reductive PET was oxidative PET. The energy difference between the quencher’s lowest unoccupied molecular orbitals (LUMO) and the highest occupied molecular orbitals (HOMO) of CQDs was what propelled reductive electron transfer. The energy difference between the LUMO of the CQDs and the LUMO of the quencher was what propelled oxidative electron transfer [83,84]. Accordingly, the PET mechanism shows that the lifetimes of CQDs are decreasing and that there is an energy gap between the HOMO and LUMO levels of the CQDs and the quencher [85]. As an example, Amira S. Gouda et al. [86] used thiosemicarbazone as a nitrogen and sulfur source and citric acid as a carbon source to create fluorescent probes for the measurement of baloxavir marboxil (BXM) using a straightforward, economical, single-stage hydrothermal method to produce extreme photoluminescence co-doped, nitrogen and sulfur CQDs. At concentrations of 10.0–100.0 ng mL^−1^, the fluorescence intensity reduction of N, S-CQDs showed a “turn-off” reaction to BXM, with a LOD of 1.88 ng mL^−1^ and a LOQ of 5.69 ng mL^−1^, respectively.

### 3.2. Fluorescence “Turn-On” Sensors

High sensitivity and ease of use are combined in fluorescence-based sensing. “Turn-on” sensors, which show an increased fluorescence signal upon analyte attachment, are frequently chosen over “turn-off” (quenching) sensors among the several fluorescence sensing techniques [87]. In CQDs, fluorescence turn-on sensing techniques are usually accomplished by reaction-based signal creation or fluorescence recovery. Reaction-based systems create new emissive centers by chemically changing surface functional groups, whereas displacement mechanisms remove coordinated quenchers to restore emissive surface states. Turn-on sensors increase analytical selectivity by being more reliable and less vulnerable to inner filter effects and nonspecific quenching than turn-off sensors [76]. Because the rise in signal is assessed against a low or dark background, turn-on systems typically offer superior sensitivity. This lowers the possibility of false-positive signals and improves the signal-to-noise ratio [88]. A “turn-on” fluorescence response can be caused by several mechanisms, such as aggregation-induced emission (AIE), disruption of Förster resonance energy transfer (FRET) to a quencher, inhibition of photoinduced electron transfer (PET), or conformational changes in the fluorophore or sensor–analyte complex that enhance radiative emission and block non-radiative decay pathways [76]. CQDs are prepared by Gopi Kalaiyarasan et al. [89] from *p*-phenylenediamine by the hydrothermal method to get the inherent optical features of pH-dependent and excitation wavelength-independent fluorescence emission. The intrinsic core of CQDs, which is linked to photoinduced electron transfer (PET), is the source of their red emission. The decrease in PET caused by the oxidation of CQDs is the cause of the turn-on fluorescence seen in the presence of cystine. Consequently, they identify cystine with a LOD of 0.4 nM and a concentration range of 10 nM to 10 μM.

### 3.3. Ratiometric Fluorescence Sensors

Because of their excellent sensitivity and stability, ratiometric fluorescence sensing techniques are frequently employed in analysis and detection [90]. Dual emission ratio fluorescence sensors, which include intrinsic carbon-core states and surface-related states brought about by functional groups or heteroatom doping, generate two fluorescence emission peaks with distinct emission wavelengths under the same excitation wavelength. These devices allow for ratiometric sensing techniques, in which one emission band preferentially reacts to analyte contact while the other serves as an internal reference [91,92]. Compared to single-emission “turn-off” systems, this self-calibrating behavior greatly improves analytical resilience, decreases vulnerability to instrumental and environmental changes, and increases analysis sensitivity and accuracy. Furthermore, it is possible to discriminate between fluorescent sensors having two fluorescence emission peaks situated in distinct emission bands and to modify the fluorescence color when exposed to ultraviolet (UV) lamp irradiation. The fluorescence color will alter in accordance with the introduction of a target analyte or variations in the target analyte’s concentration. Visual detection, or the ability to see this with the naked eye, improves the efficiency and convenience of target analysis and detection [93].

### 3.4. Environment-Sensitive and Solvatochromic CQDs

The solvatochromic fluorescence characteristic of environment-sensitive CQDs is mainly caused by surface-state emission and charge-transfer excited states [94]. Changes in the absorbance spectra of CQDs are mostly caused by variations in the surface states of produced particles with functional groups like -COOH, -OH, and -NH_2_, which allow for strong interaction with various solvents through hydrogen bonding and protonation–deprotonation equilibria [95,96]. The excited-state stabilization by the polar solvent molecules is probably the cause of the emission shift to the lower energy region with increased polarity. A red-shifted emission wavelength is the result of the dipole moment of solvents with increased solvent polarity influencing the surface electronic state [97]. CQDs are attractive platforms for pH-responsive detection and environmental sensing because of their polarity- and medium-dependent emission properties. Competing ions, fluctuating ionic strength, background absorption, and pH changes provide challenges for CQD-based detectors in complex matrices like biological and environmental samples. These elements may compromise analytical accuracy by causing inner filter effects, electrostatic shielding, or non-specific quenching [98,99]. By offering intrinsic signal normalization, dual-emission CQDs increase resilience by reducing matrix-induced intensity fluctuations. In real-sample analysis, surface engineering, heteroatom doping, and lifetime-based detection techniques further improve selectivity and dependability [98].

## 4. CQD-Based pH Sensors

### 4.1. Mechanisms of pH Sensitivity in CQDs

The drawbacks of organic dyes and inorganic QDs, such as toxicity, PL quenching, poor biocompatibility, large particle size, and background autofluorescence, are eliminated by CQDs as pH-sensitive nanoprobes [100,101]. The PL parameters of CQDs, including PL intensity, emission maxima, and excitation-dependent features, underwent significant modifications as a result of the pH condition adjustment [102]. Energy alignment between the π–π* core states and surface-trapped states is changed as a result of protonation and deprotonation of surface functional groups and heteroatoms in the structure. This, in turn, affects the PL spectra and intensity of the CQDs [65,84].

Most of the surface groups were deprotonated in alkaline circumstances. Consequently, a negative “protective shell” developed on the CQDs’ surface. The material’s great stability was demonstrated by the deprotonation of oxygen-related groups, which increased the electron density, electrostatic repulsion, accelerated radiative transitions, and created isolated particles with a high zeta potential [66,103]. Protonation, on the other hand, usually inhibits electron-donating capacity in acidic environments and can encourage non-radiative pathways. The zeta potential values also drastically decreased, indicating the breakdown of the protective shell and the creation of novel surface states. These events could lead to PL quenching, pH-dependent aggregation, and a redshift in emission maxima [10]. The band gap narrowed as a result of the CQDs’ aggregation, which produced bigger nanoparticles. The emission of green fluorescence in an acidic atmosphere could be explained by this phenomenon [103]. Thus, band-edge positions and surface energy levels alter as a result of pH-induced surface state modification, resulting in detectable changes in fluorescence behavior as shifts in emission wavelengths or intensity.

### 4.2. Fluorescent pH Sensors Based on CQDs

**Figure 2:**
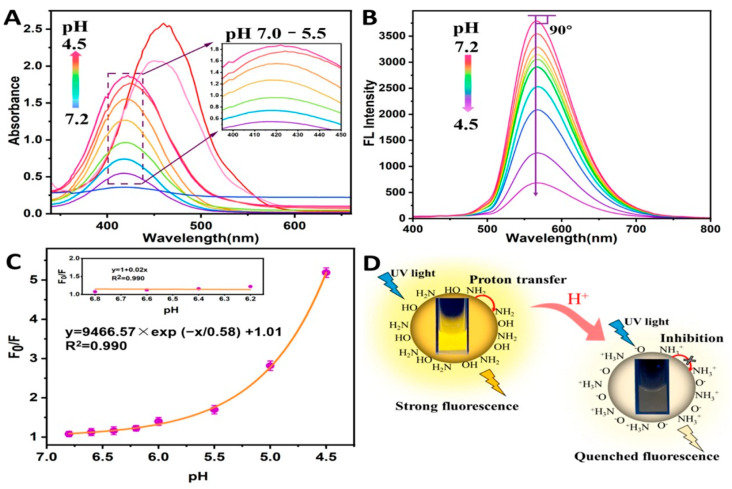
(**A**) UV-Vis absorption spectra of N-CQDs solution at various pH values (4.5–7.2); (**B**) Fluorescence spectra of N-CQDs solution at different pH values; (**C**) Exponential fitting curve between F_0_/F and pH values (7.0–4.5), inset: linear curve between F_0_/F and pH values (6.8–6.2), the error bars were equal to the standard deviation of three measurements; (**D**) Schematic illustration of fluorescence quenching of N-CQDs. Reproduced from Ref. [107]. Copyright 2022, MDPI. Licensed under CC BY 4.0.

The monotonic fluctuation of fluorescence intensity as a function of proton concentration, which is usually controlled by the protonation–deprotonation of surface functional groups and the corresponding modulation of radiative and non-radiative recombination pathways, is the basis for pH sensing in intensity-based systems. These sensors are easy to build and quite sensitive within a certain pH range, but environmental variations, probe concentration, and excitation power can all affect how accurate they are [102,103]. pH monitoring using innovative, resilient, and sensitive technologies may offer special benefits in practical applications for a range of sensing tasks, from biomedical diagnostics to environmental monitoring [104,105]. CQDs are suitable for real-time pH mapping across a wide range of chemical and biological conditions because of their small size, low cytotoxicity, and versatile surface ease of use. CQD-based fluorescent pH sensors have been used in a variety of media, including biological environments for intracellular and organelle-specific pH imaging, aqueous solutions for environmental monitoring, and heterogeneous systems like polymer matrices and nano compartments [106]. Dual-emission CQDs, in which one emission band is pH-sensitive and the other acts as an internal reference, are used in ratiometric pH sensors to get around these restrictions. A self-calibrated signal is produced by the ratio of the two emission intensities, reducing outside interference and enhancing quantitative dependability [90,98]. Hu et al. [107] designed a pH-responsive fluorescence sensor based on N-CQDs for observing milk freshness. As pH drops, the fluorescence quenching process gradually changes from dynamic to static. The suppression of H^+^ transport from N atoms to the conjugated structure is responsible for this change, as illustrated in Figure 2. When milk freshness declines in the presence of UV light, these sensors produce human-readable signals that shift from intensely yellow-green to blue.

### 4.3. CQD-Based pH Sensors in Practical and Commercial Contexts

Because of their chemical stability, reversible response, and platform adaptability, CQD-based pH sensors exhibit significant promise for translation from lab research to real-world and commercial applications. Stability under continuous irradiation, fluctuating temperature, and varying ionic strength is crucial for real-world deployment [108]. Another crucial factor is reversibility, since dependable pH sensors need to be able to withstand several cycles of protonation and deprotonation without experiencing appreciable signal drift. The fluorescence response of CQDs in well-designed systems may quickly recover when switching between basic and acidic conditions, demonstrating strong surface-state dynamics and structural integrity [109,110]. Building repeatable calibration curves over the desired operating range is necessary for accurate pH measurement.

## 5. Target Analytes and Application Domains

Sensor arrays have been shown to detect a large number of analytes [111]. Various materials, including metal oxides [112], fluorescent dyes [113], conductive polymers [114], and other nanomaterials [115,116] can be used. Although these sensing elements are highly sensitive, they have constraints in getting huge arrays of sensors for multi-analyte detection since they are expensive or may involve toxicity [117]. The features of CQDs, tunable surface groups, minimal cell toxicity, strong water solubility, ultra-small size, low cost, and ease of preparation, have garnered a lot of interest in the chemical sensing and biosensing domains [118].

### 5.1. Metal Ions

Metal ions can react with CQDs passivated with carboxyl, amino, hydroxyl, or hetero atoms to create complexes; the particular sensing element and metal ions utilized determine the variation in binding strength. Lead (Pb), arsenic (As), mercury (Hg), and cadmium (Cd) are among the extremely hazardous metals that can alter human DNA.

Lead is a non-biodegradable, hazardous transition metal that can react with blood [119,120]. It is used in many different industries, including alloys, batteries, pipes, pigments, and protective coatings [121]. Because lead is present in trace amounts in water pipes and can migrate with water due to pipe deterioration, it can be found in drinking water. It can lead to mental illnesses, memory loss, and other health problems when ingested by humans, even at very low concentrations of more than 5 mmol/L [122]. Tanmay Vyas et al. [123] have created a chemical sensor for the detection of lead in water using CQD. According to the sensing and analytical results, lead could be detected in the concentration range of 0–100 μM with a response time of 1 min and a LOD of 18.3 nM. A multitude of CQD-based fluorescent probes have been created for the detection of Pb^2+^ with elevated sensitivity and selectivity (Table 1).

Since mercury ions (Hg^2+^) have no useful function in biological systems, they are regarded as extremely hazardous pollutants. Because of their intrinsic toxicity and propensity to accumulate gradually in living organisms, these ions pose serious threats to human health and environmental integrity even at lower concentrations [124]. Organometallic compounds based on Hg–C bonds can linger in the air and water for extended periods of time, disrupt biological processes, and harm the nervous system if consumed by humans. Daraksha Bano et al. [125] synthesized CQDs utilizing Tamarindus indica leaves in a single-step hydrothermal treatment that had a high QY of roughly 46.6%, a high sensitivity to mercury ions, and a minimum LOD as low as 6 nM in the dynamic range of 0 to 0.1 μM. Nitrogen-doped high luminescent CQDs (quantum yield 40.5%) based on citric acid monohydrate and ammonia were made using a one-step hydrothermal method and used as Hg^2+^ sensor with a LOD of 0.087 µM. They can be used in a natural water sample with a recovery between 96.6 and 105.5% [126]. Other studies are illustrated in Table 1.

An extremely poisonous heavy metal, arsenic, poses serious health concerns to humans. It can cause disorders like diabetes, birth defects, liver and kidney damage, arsenicosis, hemolysis, cancer, neurological problems, and painful skin lesions [127]. Md Bayazeed Alam et al. [128] created a chlorophyll-functionalized CQD as a nanoprobe for the sensitive detection of the As^3+^ ion in aqueous solutions by hydrothermally treating banana leaf extract in a single step. They used concentrations of 10 µM and 100 µM to show that the intensity increases with the addition of As^3+^. Sweta Rani et al. [129] employed manganese, nitrogen, and sulfur as multimodal CQD dopants to detect arsenic ions with a LOD of 0.22 ppb. Saikia et al. [130] created a hybrid of polyaniline nanofiber and CQDs for the fluorometric detection of As^3+^ with great sensitivity. At the maximum concentration, the fluorescence response to As^3+^ significantly increased by 9.2 times, rising from 0.001 ppb to 2.000 ppb. The technique demonstrated its efficacy in monitoring trace levels of As^3+^ in contaminated water by achieving a LOQ of 2.052 ppb and a LOD of 0.677 ppb.

One dangerous soft element is cadmium. Its pollutants, which harm the kidneys and lungs, can be created by natural deposit erosion, paint and battery waste runoff, and metal refinery discharge [131]. Qiushuang Ai et al. [132] produced CQDs for Cd^+2^ sensing using a hydrothermal method using citric acid and triethyl amine, with a range of 0.1 μM to 23 μM and a LOD of 0.018 μM.

**Figure 3:**
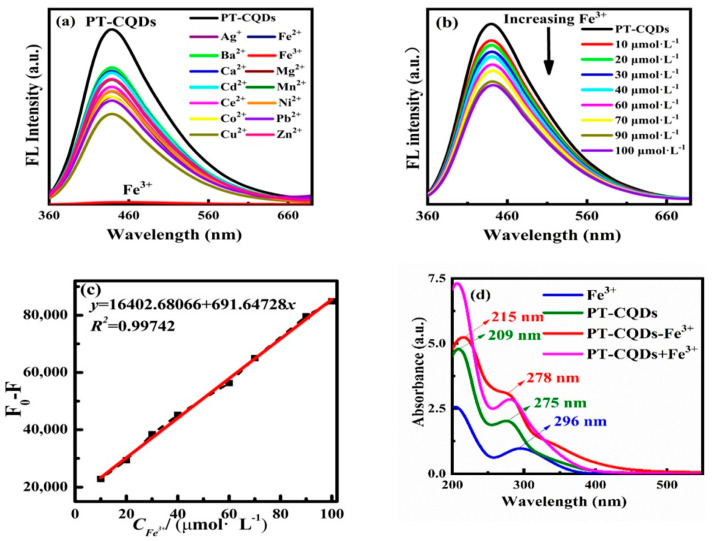
(**a**) Fluorescence spectra of PT-CQDs at the emission wavelength of 440 nm with various metal ions. (**b**) PT-CQD fluorescence spectra with varying Fe^3+^ ion concentrations. (**c**) Fluorescence intensity changes linearly with Fe^3+^ ion concentration. (**d**) The UV-visible absorption spectra of PT-CQDs, Fe^3+^, PT-CQDs-Fe^3+^ and PT-CQDs + Fe^3+^ solution. Reproduced from Ref. [137]. Copyright 2025, MDPI. Licensed under CC BY 4.0.

Up to a certain point, iron (III), one of the most widely used metal ions, is necessary for human health because it is frequently present in drinking water and municipal wastewater. Beyond that point, however, it can lead to conditions like diabetes, inflammation, Alzheimer’s disease, and more. Additionally, anemia (IDA) can result from an iron deficiency in the body [133]. Monitoring the amount of Fe^3+^ in the environment, and more especially in patients with diseases caused by iron (III), is crucial since iron (III) in the environment also affects plant growth [134]. Fe^2+^ ions are also found in nature, are vital to human health, and need to be observed, yet they are hard to detect precisely because they oxidize to Fe^3+^ in an open environment. So, there is very little research on Fe^2+^ detection [135]. Fe^3+^ induced fluorescence quenching of CQDs is mainly caused by strong interactions between Fe^3+^ ions and surface groups on the CQDs that contain oxygen or nitrogen, such as carboxyl, hydroxyl, and especially phenolic –OH functions. Through inner-sphere coordination, these groups can chelate Fe^3+^, offering a polar environment that promotes complexation and numerous oxygen donor sites [134]. Several studies (Table 1) have reported CQD-based nanosensors for Fe^3+^ detection with low detection limits and good selectivity. For example, Pei Zhao et al. [136] employed the hydrothermal process to create CQDs from water hyacinth. After obtaining CQDs of uniform size, they used them to detect Fe^3+^ and found a LOD of 0.084 μM, which is even lower than the 0.77 μM set by the World Health Organization. Lina Zhong et al. [137] prepared CQDs from Peperomia tetraphylla using a hydrothermal process, which are used as a fluorescence sensor because of their great sensitivity and selectivity to ferric ions (Fe^3+^). The World Health Organization’s maximum allowable level of Fe^3+^ ions in drinking water (5.36 μmol·L^−1^) is higher than the LOD, which was 2.7 μmol·L^−1^. As the concentration of Fe^3+^ ions increases, the fluorescence intensity of PT-CQDs steadily decreases throughout the range of 0~100 μmol·L^−1^, as shown in Figure 3.

Industrial effluent contains chromium, a hard metal that is extremely hazardous. Trivalent chromium Cr (III), which is non-toxic at low quantities, and hexavalent chromium Cr (VI), which is extremely dangerous even at low concentrations, are its two main oxidation states. It is advised that the amount of Cr (VI) in drinking water be less than 100 parts per billion because it might cause cancer and hormonal issues if ingested in significant proportions by people. Qianqian Huang et al. [138] reported CQDs made from the polysaccharide of Poria cocos. These CQDs have a LOD of 0.25 mM for Cr (VI). The excitation spectra of CQDs overlap with the absorption spectra of Cr (VI), causing the excitation light of CQDs to be absorbed by Cr (VI), resulting in the inner filter effect, which quenches CQD fluorescence. The zeta potential after mixing is also increased from −18.8 to −7.06 mV due to the binding of the functional group with Cr (VI).

Copper (Cu^2+^) is a borderline ion that is necessary for the healthy growth of biological activity since it builds the bones and immune system; nevertheless, an excessive amount of Cu^2+^ can induce sickness, discomfort, and disruption of biological activity [139]. As a result, it is required to design simple and inexpensive sensing technology for detecting Cu^2+^. Xiaofeng Wei et al. [140] prepared N-doped CQDs in a hydrothermal method using ammonium citrate and ethylene diamine as precursors and passivated with metal -organic framework, achieving a broad detection range from 5 × 10^−8^ to 1 × 10^−4^ M and a remarkable LOD of 1.44 × 10^−8^ M.

A significant number of CQD-based fluorescence sensors have been documented for the detection of diverse metal ions, including Hg^2+^, Pb^2+^, Cd^2+^, Cr^6+^, Cu^2+^ and Fe^3+^, exhibiting exceptional analytical performance (Table 1). The table is categorized by analyte, with studies arranged sequentially; studies testing numerous analytes are listed in a unified order to avoid repetition.

**Table 1:** Comparative investigation of metal ion detection utilizing CQDs prepared from various precursors.

Precursors	Synthesis Conditions	Method	Ions	Ions That Do Not Interfere	LOD (μM)	Ref.
Ice apple	180 °C for 12 h	Hydrothermal	Fe^3+^	K^+^, Fe^2+^, Na^+^, Pb^2+^, Co^2+^, Zn^2+^, Ca^2+^, Hg^2+^, Cd^2+^, Cr^2+^, Cu^2+^, Al^3+^	2.01	[141]
Hot peppers	180 °C for 24 h	Hydrothermal	Fe^3+^	Mn^2+^, Al^3+^, Fe^+2^, Hg^2+^, Cu^2+^	1	[142]
Citric acid and Ethylenediamine	200 °C for 5 h	Hydrothermal	Fe^3+^	Na^+^, K^+^, Ni^2+^, Ca^2+^, Co^2+^, Sn^2+^	0.07	[143]
Citric acid and Phenylalanine	180 °C for 30 h	Hydrothermal	Fe^3+^	Ca^2+^, Cd^2+^, Co^2+^, Cr^3+^, Cu^2+^, Mg^2+^, Pb^2+^ Zn^2+^	0.720	[144]
Citric acid, Urea and Borax	180 °C for 10 h.	Hydrothermal	Fe^3+^	Mg^2+^, Pb^2+^, Ca^2+^, Cu^2+^, Mn^2+^, Ni^2+^, Hg^2+^, Ba^2+^, Cd^2+^, Co^2+^, Al^3+^, Na^+^, Ag^+^	0.044	[145]
Glucose	180 °C for 1 h	Solvothermal H_2_SO_4_	Fe^3+^	Al^3+^, Zn^2+^, Cd^2+^, Hg^2+^, Cu^2+^, Ni^2+^, Fe^2+^, Mn^2+^, Cr^2+^, Co^2+^, Mg^2+^,	0.002	[146]
Simmondsia (jojoba) leaves	180 °C for 10 h	Hydrothermal	Fe^3+^	Ca^2+^, Co^2+^, Cu^2+^, Mg^2+^, Mn^2+^, Ni^2+^, Zn^2+^	0.018	[134]
Xylan and amikacin sulfate	240 °C for 18 h	Solvothermal NaOH/urea	Fe^3+^	Mn^2+^, K^+^, Al^3+^, Pb^2+^, Cr_2_O_7_^2−^, Cd^2+^, Ag^+^, Ba^2+^, Cl^−^, NO_3_^−^, CH_3_COO^−^, SO_4_^2−^	0.198	[147]
Roasted chickpeas	Microwave at 350 watts for 2 min	Microwave- assisted pyrolysis	Fe^3+^	Na^+^, K^+^, Mg^2+^,Ca^2+^, Sr^2+^, Ba^2+^, Be^2+^, Se^2+^, Cr^3+^, Mn^2+^, Co^2+^, Ni^2+^, Cu^2+^, Zn^2+^, Ag^+^, Cd^2+^, Au^3+^, Hg^2+^, Al^3+^, Pb^2+^, Bi^3+^, B^+^, Sn^2+^, As^2+^, Pd^2+^, Tl^+^, Y^3+^, Sc ^3+^, V^3+^ W^2+^, Mo^2+^, Ti^4+^	2.74	[148]
Magnolia flower	200 °C for 8 h	Hydrothermal	Fe^3+^	K^+^, Na^+^, Pb^2+^, Fe^2+^, Zn^2+^, Cu^2+^, Mg^2+^, Ca^2+^, Al^3+^, Cd^2+^, Cr^3+^, Ba^2+^, Hg^2+^	0.073	[149]
Carbon rods	180 °C for 12 h	Hydrothermal/ solvothermal ammonia	Cr^6+^	Al^3+^, Mg^2+^, Mn^2+^, Li^+^, K^+^, Sb^3+^, Cd^2+^, Zn^2+^, Hg^2+^, Fe^2+^	0.242	[150]
			Cu^2+^		0.251	
			Fe^3+^		0.161	
Salicylic acid	200 °C for 4 h	Hydrothermal pyrolysis	Fe^3+^	Co^2+^, Cd^2+^, Cu^2+^, Ca^2+^, Ba^2+^, Mg^2+^, Zn^2+^, Mn^2+^, K^+^, Al^3+^, Na^+^, Hg^2+^, Pb^2+^, Fe^2+^, Ag^+^	0.052	[151]
Composite of CQDs, ZnO, and CdS	200 °C for 5 h	Precipitation	Fe^3+^	Ba^2+^, Ca^2+^, Cu^2+^, K^+^, Mg^2+^, Na^+^, Ni^2+^, NH_4_^+^, Mn^2+^, Ag^+^, Fe^2+^, Hg^2+^.	0.172	[152]
Tartaric acid and L-arginine	170 °C for 6 h	Solvothermal	Hg^2+^	K^+^, Li^+^, Mg^2+^, Ni^2+^, Pb^2+^, Sr^2+^, Zn^2+^	0.017	[153]
			Fe^3+^		0.50	
Citric acid and amino acids	180 °C for 9 h	Hydrothermal	Fe^3+^	Ca^2+^, Cu^2+^, K^+^, Hg^2+^, Mg^2+^, Al^3+^, Mn^2+^, Na^+^	10^6^	[154]
Triphenylamine aldehyde and 2,3-diaminopyridine	240 °C for 8 h	Solid state synthesis	Fe^3+^	K^+^, Fe^2+^, Ni^2+^, Zn^2+^, Cu^2+^, Co^2+^, Hg^2+^, Cd^2+^, Ca^2+^, Pb^2+^ Cr^3+^, Al^3+^, OAc^−^, S^2−^, CrO_4_^2−^, NO_3_^−^, NO_2_^−^, CO_3_^2−^, CN^−^, H_2_PO_4_^−^, citric acid, ascorbic acid, glucose, fructose	0.3	[155]
Chitosan and tartaric acid	200 °C for 5 h	Hydrothermal	Fe^3+^	K^+^, Na^+^, Ag^+^, Ca^2+^, Mg^2+^, Zn^2+^, Fe^2+^, Cu^2+^, Ni^2+^, Co^2+^, Ba^2+^, Mn^2+^, Cd^2+^, Pb^2+^, Al^3+^, Cr^3+^	1.2	[156]
Chitosan and EDTA	200 °C for 11 h	Hydrothermal	Fe^3+^	Pb^2+^, Li^+^, Mg^2+^, Ca^2+^, Ce^3+^, Mn^2+^, Fe^2+^, Na^+^, Zn^2+^, K^+^, Co^2+^, La^3+^, Ba^2+^, Sn^2+^, Ni^2+^, Cu^2+^	0.3	[157]
licorice powder and p-phenylenediamine	180 °C for 10 h	Hydrothermal	Fe^3+^	Fe^2+^, Cu^2+^, Zn^2+^, Mn^2+^, Ni^2+^, Co^2+^, Pb^2+^, Cd^2+^, Hg^2+^, Al^3+^, SO_4_^2−^, NO_3_^−^	0.346	[158]
Radix Vladimiriae	Microwave at 900 W for 10 min	Fast one-step microwave method	Fe^3+^	F^−^, Cl^−^, Br^−^, I^−^, CO_3_^2–^, SO_4_^2−^, Ag^+^, Ba^2+^, Zn^2+^, Co^2+^, Ni^2+^, Mg^2+^, Mn^2+^, Pb^2+^, Cu^2+^	0.062	[159]
Iranian seedless barberry	150 °C for 1 h	Hydrothermal	Fe^3+^	Co^2+^, Cu^2+^, Cr^3+^, Hg^2+^, Zn^2+^, Cd^2+^, Pb^2+^	1.73	[160]
Hedyotis diffusa willd	120 °C for 6 h	Hydrothermal	Fe^3+^	Al^3+^, K^+^, Sn^2+^, Ba^2+^, Ca^2+^, Na^+^, NH_4_^+^, Co^2+^, Cu^2+^, Mg^2+^, Zn^2+^, C_2_H_2_O_2_^−^, PO_4_^3−^, F^−^, NO_3_^−^, IO_3_^−^, Cl^−^, H_2_PO_4_^−^, HPO_4_^2−^, CN^−^, TC	6 × 10^−3^	[161]
Gardenia jasminoides	180 °C for 6 h	Hydrothermal	Fe^3+^	K^+^, Na^+^, Pb^2+^, Co^2+^, Zn^2+^, Ca^2+^, NH_4_^+^, Cu^2+^, Mg^2+^	0.29	[162]
Gelatin	200 °C for 3 h	Hydrothermal	Fe^3+^	Bi^2+^, Cd^2+^, Co^2+^, Pb^2+^, Mg^2+^, Mn^2+^, Ni^2+^, Sn^2+^, Sr^2+^, Na^2+^, Zn^2+^, Al^3+^	0.2	[163]
Cynodon dactylon biomass	Microwave at 800 W for 5 min	Microwave- assisted method	Fe^3+^	Mg^2+^, Zn^2+^, Co^2+^, Mn^2+^, Hg^2+^, Cu^2+^, Pb^2+^	0.10	[164]
			As^3+^		0.019	
Platanus acerifolia leaves	150 °C for 3 h	Carbonization and hydrothermal method	Pb^2+^	Ca^2+^, Mg^2+^ and K^+^	4 × 10^−5^	[165]
Citric acid, ethylenediamine, boric acid	150 °C for 72 h	Solvothermal Ethanol	Pb^2+^	Ni^2+^, Cu^2+^, Cd^2+^, Mn^2+^, Zn^2+^, Co^2+^, Na^+^, Li^+^	5 × 10^−3^	[166]
Watermelon juice	160 °C for 16 h	Hydrothermal	Pb^2+^	Li^+^, Na^+,^ Mg^2+^, Al^3+^, K^+^, Ca^2+,^ Mn^2+^, Fe^2+,^ Co^2+,^ Ni^2+^, Cu+^2^, Zn^2+^, Cd^2+^, In^2+^, Cs^+^, Ba^2+^	2 × 10^−4^	[167]
Citric acid, glutamine, and sodium sulphide	200 °C for 3 h	hydrothermal	Pb^2+^	Co^2+^, Cd^2+^, Fe^3+^, Cr ^3+^, Zn^2+^, Na^+^, K^+^ Cl^−^, Br^−^, I^−^, CO_3_^2−^, PO_4_^−^, NO_3_^−^	0.0135	[168]
R. ribes powder	200 °C for 10 h	Hydrothermal	Pb^2+^	Cr^3+^, Zn^2+^, Fe^2+^, Ca^2+^, Na^1+^, Mg^2+^, Ni^2+^, Cu^2+^, Cd^2+^, As^3+^	0.047	[169]
Walnut green skin, Acetamide Glycolic Acid	220 °C for 10 h	Hydrothermal	Pb^2+^	Na^+^, Mg^2+^, Ca^2+^, Zn^2+^, Fe^3+^, K^+^, Cu^2+^, Co^2+^	0.0015	[170]
Fenugreek seeds	180 °C for 8 h	Hydrothermal	Pb^2+^	Al^3+^, Ca^2+^, Cd^2+^, Cr^3+^, Co^2+^, Cu^2+^, Cu^+^, Fe^2+^, Fe^3+^, K^+^, Sn^4+^, Na^+^, Ni^2+^, Mn^2+^, Zn^2+^, Sr^2+^, (NH_4_)_6_Mo_7_O_24_, Cr^6+^, Sb^3+^, Ba^2+^, Li^+^, Mg^2+^	9.345	[171]
L-cysteine	160 °C for 10 h	Hydrothermal	Pb^2+^	Cu^2+^, Fe^3+^, Al^3+^, Cd^2+^, Mn^2+^, Ca^2+^, Na^+^, Zn^2+^, La^3+^, K^+^, Cr^3+^	0.45	[172]
o-phenylenediamine (OPD) and taurine	200 °C for 8 h	Hydrothermal	Hg^2+^	Ag^+^, Ba^2+^, Ca^2+^, Co^2+^, Cr^3+^, Cu^2+^, Fe^2+^, Fe^3+^, K^+^, Mg^2+^, Mn^2+^, Na^+^, Pb^2+^, Zn^2+^	0.0450	[173]
2,3-diaminopyridine and citric acid	220 °C for 10 h	Hydrothermal	Hg^2+^	Na^+^, K^+^, Ca^2+^, Mg^2+^, Fe^3+^, Ni^2+^, Zn^2+^, Cu^2+^, Cd^2+^, Ag^+^, NO_3_^−^, SO_4_^2−^, Cl^−^	0.0424	[174]
Succinic Acid and l-cysteine	205 °C for 210 min	Hydrothermal	Hg^2+^	Pb^2+^, Cr^3+^, Ag^+^, Co^2+^, Cd^2+^, Fe^2+^/Fe^3+^, Cu^2+^, Zn^2+^, Ni^2+^, Ca^2+^, Mg^2+^	0.237	[175]
Citric acid and Urea	microwave for 240 s	Microprocessor- assisted synthesis	Hg^2+^	Mg^2+^, Ca^2+^, Mn^2+^, Fe^2+^, Co^2+^, Ni^2+^, Cu^2+^, Zn^2+^, Cd^2+^, Pb^2+^, Al^3+^, Fe^3+^, Na^+^	0.0018	[176]
Pomegranate peel	220 °C for 12 h	Hydrothermal	Hg^2+^	Mn^2+^, Cu^2+^, Co^2+^, Pb^2+^, Ni^2+^, Zn^2+^, Ca^2+^, As^3+^, Fe^3+^, Na^+^, K^+^, Mg^2+^, Ag^+^	0.0005	[177]
Citric acid and urea	microwave 700 W for 5 min	Microwave method	Hg^2+^	Na^+^, Cu^2+^, Fe^2+^, Ag^+^, K^+^, Zn^2+^, Ba^2+^	0.080	[178]
Polyethylene glycol and tryptophan	180 °C for 12 h	Hydrothermal	Hg^2+^	Li^+^, Na^+^, K^+^, Ag^+^, Ca^2+^, Mg^2+^, Cd^2+^, Mn^2+^, Co^2+^, Ni^2+^, Zn^2+^, Al^3+^, Fe^3+^, Sn^4+^, Pb^2+^, Cu^2+^, Cr^3+^, O_2_^∙−^, H_2_O_2_, NO, H_2_S, ONOO^−^, ClO^−^, SO_3_^2−^	0.0063	[179]
Glucose, PEI and Gleditsia sinensis powder	180 °C for 3 h	Calcination	Cd^2+^	Cr^3+^, K^+^, Mg^2+^, Na^+^, Pb^2+^, Hg^2+^, Fe^3+^, Rb^+^, Cs^+^, Ca^2+^, Cu^2+^ PO_4_^3−^, HSO_4_^−^, SO_4_^2−^, Cl^−^, I^−^, NO_3_^−^, CO_3_^2−^, Br^−^	4.05	[180]
Ammonium citrate and glutamic acid	180 °C for 8 h	Hydrothermal	Cd^2+^	Ba^2+^, Ca^2+^, Hg^2+^, Mn^2+^, Fe^3+^, Ag^+^, Mg^2+^, Pb^2+^, K^+^, Cu^2+^, Cr^3+^	0.013	[181]
Fish scale wastes	160 °C for 18 h	Hydrothermal	Cd^2+^	Hg^2+^, Fe^2+^, Cu^2+^, Ni^2+^	-----	[182]
Citric acid and triethylamine	160 °C for 6 h	Hydrothermal	Cd^2+^	Na^+^, K^+^, Ca^2+^, Mg^2+^, Zn^2+^, Fe^3+^, Cu^2+^, Ba^2+^, Pb^2+^ and Mn^2+^	0.018	[132]
Water amaranth leaves	180 °C for 5 h	Hydrothermal	Cd^2+^	Mn^2+^, Cr^3+^, Cs^+^, Hg^2+^, Ba^2+^, Mg^2+^, Fe^2+^, Fe^3+^, K^+^, Na^+^, Pb^2+^	0.015	[183]
Citric acid, pyridine, sodium borohydride	180 °C for 150 min	Hydrothermal	Cd^2+^	Zn^2+^, K^+^, Na^+^, Ag^+^, Ba^2+^, Mg^2+^, Mn^2+^, Ca^2+^, Cr^6+^, Co^2+^ and Cr^3+^	0.28	[184]
Syzygium aromaticum	300 °C for 2 h	Carbonization- assisted ultrasonication	Fe^3+^	Ni^2+^, Cu^2+^, Fe^2+^, Co^2+^, Bi^3+^, Pb^2+^, Hg^2+^, Ag^+^, Sn^2+^, Zn^2+^, and Cr^3+^	0.515	[185]
			Cd^2+^		0.403	
Natural precursor apricot	150 °C for 3 h	Hydrothermal	Cr^6+^	Cu^2+^, Ni^2+^, Zn^2+^, Mn^2+^, Mg^2+^, Fe^2+^, Fe^3+^, Ba^2+^, Hg^2+^, Pb^2+^, Cd^2+^, Cr^3+^, Co^3+^, As^3+^	2.07	[186]
Ammonium citrate and lactose	180 °C for 4 h	Hydrothermal	Cr^6+^	Cu^2+^, Ca^2+^, Zn^2+^, Fe^2+^, Mg^2+^	0.767	[187]
β-cyclodextrin	180 °C for 12 h	Hydrothermal	Cr^6+^	Ag^+^, Ca^2+^, Cu^2+^, Fe^2+^, Fe^3+^, Hg^2+^, K^+^, Mg^2+^, Mn^2+^, Na^+^, Pb^2+^, Cd^2+^	0.213	[188]
Houttuynia cordata	200 °C for 9 h	Hydrothermal	Cr^6+^	Ba^2+^, Fe^3+^, Na^+^, Cu^2+^, Mg^2+^, Co^2+^, Fe^2+^, Zn^2+^, Pb^2+^, Mn^2+^, K^+^, Cd^2+^, Hg^2+^, Al^3+^	0.163	[189]
Cumin seeds	215 °C for 5 h	Hydrothermal	Cr^6+^	Ca^2+^, Ag^2+^, Cd^2+^, Ni^2+^, Hg^2+^, Al^3+^, Pb^2+^, K^+^, Cu^2+^, Sn^2+^, Co^2+^, Ti^4+^, Zn^2+^, As^5+^	8.3	[190]
Wool keratin and ethylenediamine	180 °C for 10 h	Hydrothermal	Cr^6+^	Ni^2+^, Pb^2+^, Ag^+^, Ca^2+^, Mn^2+^, Fe^3+^, Co^2+^, Mg^2+^, Al^3+^, Cr^3+^, SO_4_^2−^, NO_3_^−^, NO_2_^−^, NH_4_^+^	0.011	[191]
Lycium barbarum	180 °C for 8 h	Hydrothermal	Cr^6+^	K^+^, Na^+^, Ca^2+^, Ag^2+^, Cu^2+^, Zn^2+^, Mg^2+^, Al^3+^, Cd^3+^, SO_4_^2−^, Cl^−^, NO_2_^−^, NO_3_^−^, F^−^, CH_3_COO^−^, PO_4_^3−^, BO_4_^−^	0.16	[192]
Ammonium citrate and ethylenediamine	heated for 2 min in a microwave	Microwave-assisted pyrolysis	Cu^2+^	Biosensor with reduced interference	0.0195	[193]
Citric acid	150 °C for 5 h	Hydrothermal	Cu^2+^	Cr^2+^, Cd^2+^, Ca^2+^, Pb^2+^, Mg^2+^, Zn^2+^, Sr^2+^, Rb^+^	0.252	[194]
Carbamide and Gallic acid	microwave oven for 10 min	Microwave	Cu^2+^	Co^2+^, Mn^2+^, Ag^+^, Cs^2+^, Zn^2+^, Hg^2+^, Na^+^, Fe^2+^, K^+^, Mg^2+^, Ba^2+^, Ni^2+^, Ca^2+^, Al^3+^, Fe^3+^	0.0005	[195]
Trachyspermum ammi seeds	195 °C for 200 min.	Hydrothermal	Cu^2+^	Cd^2+^, Hg^2+^, Ag^+^, As^5+^, Ni^2+^, Pb^2+^, Zn^2+^, Al^3+^, Ca^2+^, K^+^, Sn^2+^, Ti^4+^	1.01	[196]
L-cystiene	5 s at 135 °C in a microwave	Microwave- assisted hydrothermal method	Cu^2+^	Zn^2+^, Mg^2+^, Ca^+^, K^+^, Ba^2+^, Na^+^, Hg^2+^, NH^4+^, Mn^2+^, Fe^2+,^ Fe^3+^, Ni^2+^, Li^+^, Co^2+^, Cd^2+^, Pb^2+^, Ag^+^, Sr^2+^	0.0025	[197]
Ammonium citrate and triethylenetetramine	2 min in a microwave	Microwave-assisted pyrolysis	Cu^2+^	Ag^+^, Al^3+^, Ca^2+^, Cd^2+^, Co^2+^, Cr^2+^, Fe^2+^, Fe^3+^, Hg^2+^, K^+^, Mg^2+^, Mn+, Na^+^, Ni^2+^, Pb^2+^, Zn^2+^	0.0045	[198]

#### Discussion of Metal Ion Sensing Mechanisms Using CQDs

In CQD-based ion sensing, selectivity is typically discussed only through “positive” outcomes (the detected analyte and its LOD), which creates a survivorship-bias-type blind spot. A useful inversion of perspective is to treat the routinely reported “ions that do not interfere” as explicit negative tests: these ions were experimentally challenged and did not elicit a comparable fluorescence response under the same CQD system. This is analogous to Abraham Wald’s classic WWII analysis of aircraft damage: the least “hit” regions on returning aircraft were not safe, but rather fatal when hit, because those aircraft did not return. Likewise, ions repeatedly tested as non-interfering yet seldom reported as detected define a “negative chemical space” of CQD fluorescence modulation.

In our dataset, several ions (e.g., Zn (II), Mg (II), Ca (II), Na (I), K (I)) appear dozens of times as non-interfering controls while being absent as detected targets, suggesting intrinsic mechanistic limitations of conventional CQD sensing pathways for redox-inert, closed-shell or weakly quenching ions, independent of how frequently they are tested.

To visualize this negative chemical space, each ion reported in the dataset was treated as an experimental event with two possible outcomes:

Detected (positive fluorescence response under defined conditions),

Non-interfering (explicitly tested but did not produce a significant response).

For every ion, we calculated:

Tested_total = number of explicit challenges (detected + reported as non-interfering),

Failure_ratio = non-interfering/tested_total.

Only ions with Tested_total > 10 were included to ensure statistical relevance and avoid artifacts from single-case reports.

Ions with a high failure ratio were repeatedly tested yet never reported as detected targets in this dataset. These include Zn (II), Mg (II), Ca (II), Na (I), K (I), Co (II), Mn (II), Ni (II), Fe (II), and Al (III). In contrast, Fe (III) shows a markedly lower failure ratio, indicating that it is frequently detected relative to the number of times it is challenged.

The relation, therefore, does not show “which ions are best detected” but rather reveals which ions are structurally resistant to CQD fluorescence modulation across diverse synthetic strategies and precursor chemistries.

This inversion of perspective transforms routine selectivity tables into a statistically interpretable map of mechanistic limitations.

The negative chemical space revealed is not random. When interpreted in the context of precursor-derived surface chemistry and hard–soft acid–base (HSAB) principles, a coherent mechanistic pattern emerges.

CQDs synthesized from oxygen-rich precursors such as carbohydrates or organic acids predominantly expose hydroxyl, carbonyl, carboxylate, and lactone functionalities. The introduction of nitrogen-containing reagents (e.g., urea, ethylenediamine, melamine) generates amine, amide, and heterocyclic nitrogen sites, whereas sulfur-containing precursors (e.g., thiourea, cysteine, biomass-derived sulfur species) may introduce thiol or thioether functionalities. Thus, the precursor determines the distribution of hard (O-donor), borderline (N-donor), and soft (S-donor) surface coordination sites.

From a classical HSAB perspective, hard acids such as Fe (III), Al (III), Mg (II), and Ca (II) preferentially bind to oxygen-containing functional groups, while soft acids such as Hg (II) favor sulfur-containing ligands. Borderline acids such as Cu (II), Pb (II), Ni (II), and Zn (II) exhibit intermediate behavior. However, the statistical analysis of detection outcomes indicates that HSAB complementarity alone does not guarantee fluorescence modulation.

For example, Zn (II) is frequently classified as a hard or borderline acid and would be expected to interact with oxygen-rich CQD surfaces. Nevertheless, it appears consistently within the negative chemical space: it is repeatedly tested yet rarely detected as a primary analyte. Similarly, Mg (II) and Ca (II), although classical hard acids, show near-universal non-responsiveness across diverse CQD systems. These ions are redox-inert, closed-shell, and lack strong quenching pathways, suggesting that mere coordination is insufficient to induce measurable fluorescence change.

In contrast, Fe (III), although also a hard acid, is consistently detected. This difference is likely rooted not only in Lewis acidity but in additional mechanistic factors: strong ligand-to-metal charge transfer (LMCT), high redox activity, and efficient fluorescence quenching pathways. Likewise, Hg (II), a soft acid, benefits from heavy-atom effects and strong affinity for sulfur-doped surfaces, particularly when S-containing precursors are used. Borderline redox-active ions such as Cu (II) similarly combine coordination with paramagnetism and electron-transfer capability, enhancing their detectability.

Therefore, the emergence of a negative chemical space reflects a mechanistic hierarchy:

Lewis acid–base complementarity is necessary but not sufficient.

Effective fluorescence modulation requires additional electronic factors such as redox activity, paramagnetism, or heavy-atom-induced non-radiative decay.

Precursor chemistry controls the availability and hardness of donor sites, thereby modulating which classes of acids can interact, but the final sensing outcome depends on whether this interaction perturbs the emissive states.

In this framework, CQDs synthesized from purely oxygenated “green” precursors are biased toward hard-acid coordination but may fail to detect redox-inert hard cations. Introduction of nitrogen functionality broadens borderline interactions, while sulfur doping selectively enhances affinity toward soft acids such as Hg (II). However, even optimized donor–acceptor matching does not overcome the intrinsic fluorescence resistance of certain closed-shell ions.

Thus, the negative chemical space is not merely a statistical artifact; it encodes mechanistic limitations of CQD fluorescence sensing and reveals that ion detectability is governed by a coupled system of precursor-defined surface chemistry and ion electronic structure, rather than by HSAB classification alone.

**Figure 4:**
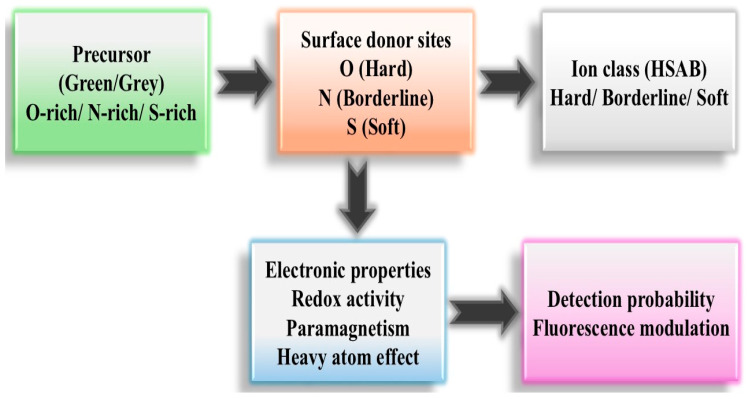
Mechanistic framework linking precursor chemistry to CQD ion detection.

The mechanistic framework proposed in Figure 4 integrates precursor chemistry, HSAB interactions, and electronic effects into a unified interpretation of CQD ion sensing. The starting point is the precursor, which determines the surface donor distribution after carbonization. Oxygen-rich precursors generate predominantly hard donor sites (hydroxyl, carbonyl, carboxylate), nitrogen-containing reagents introduce borderline donor functionalities (amines, amides, heterocycles), while sulfur-containing sources contribute soft donor sites (thiols, thioethers).

The surface donor groups regulate the principal Lewis acid-base interactions with metal ions based on HSAB principles. Nonetheless, the negative chemical space analysis suggests that donor–acceptor complementarity alone is unable to induce fluorescence modulation. The shift from coordination to a quantifiable sensory response necessitates supplementary electronic aspects, including redox activity, paramagnetism, or nonradiative pathways caused by heavy atoms.

Thus, Figure 4 illustrates that ion detectability emerges from a hierarchical process. Precursor-defined surface chemistry sets the interaction window (hard, borderline, or soft coordination), but the final sensing outcome depends on whether the ion can perturb the emissive states through charge transfer, electron exchange, or spin-related quenching mechanisms. This explains why certain hard acids such as Fe (III) are consistently detected, while other hard or borderline ions such as Zn (II), Mg (II), or Ca (II) remain in the negative chemical space despite frequent testing.

The framework, therefore, reconciles statistical observations with chemical principles: CQD sensing is controlled not by HSAB matching alone, but by the coupling between surface donor structure and the electronic structure of the ion.

The precursor determines the distribution of surface donor sites (O-, N-, or S-containing groups), which interact with metal ions according to HSAB principles (hard, borderline, soft acids). However, successful fluorescence modulation requires additional electronic factors such as redox activity, paramagnetism, or heavy-atom effects. The final detection probability, therefore, results from the coupling between precursor-derived surface chemistry and the electronic properties of the ion.

An important mechanistic nuance emerges when considering highly oxidizing ions such as Au (III). With one of the highest standard reduction potentials among commonly tested metal ions (Au^3+^/Au^0^), Au (III) would be expected to strongly interact with electron-rich carbon nanostructures. Indeed, it has been demonstrated that hydrothermally treated graphene oxide can reduce Au (III) to metallic Au (0), reflecting the substantial reducing capability of partially restored sp^2^ carbon domains and residual surface functionalities.

However, in the compiled CQD sensing dataset, Au (III) does not emerge as a robustly detected analyte. This apparent contradiction highlights a critical distinction between thermodynamic redox feasibility and fluorescence-based sensing response.

Freshly synthesized CQDs, particularly those obtained under hydrothermal conditions, may initially possess a relatively high reducing potential due to:

partially restored conjugated domains,

residual defects,

labile oxygen functionalities,

high electron density in surface states.

Such systems could, in principle, reduce Au (III) to Au (0), leading to nanoparticle nucleation rather than controlled fluorescence modulation. In this scenario, the dominant process is chemical transformation, not reversible quenching.

Over time, however, CQD surfaces evolve. Surface oxidation, reorganization of functional groups, and slow equilibration processes progressively modify the electronic structure. This evolution may be understood in the context of Ostwald-type cascades, where kinetically accessible, metastable surface states form initially and are gradually replaced by more thermodynamically stable oxygen-containing groups. The final equilibrium surface may be chemically less reducing but more oxidized and structurally stabilized.

Crucially, most sensing studies do not report the time elapsed between synthesis and ion-detection experiments. As a result, the effective redox potential and surface donor distribution of the CQDs at the time of measurement remain unknown. The observed sensing behavior, therefore, reflects not a single, well-defined material, but a time-evolved surface state.

This temporal dimension introduces an additional layer of uncertainty into the interpretation of the negative chemical space. Ions such as Au (III), despite their strong redox character, may not appear as detected analytes because:

The interaction leads to irreversible reduction rather than fluorescence modulation.

The CQDs have already undergone partial oxidation, reducing their effective electron-donor capability.

The dominant mechanism is nanoparticle formation or surface reconstruction rather than controlled electronic quenching.

Thus, the apparent absence of Au (III) in the detection landscape does not contradict its redox strength; rather, it underscores that CQD-based sensing is governed by a delicate balance between surface redox activity, kinetic evolution of functional groups, and the mechanistic pathway required to perturb emissive states.

For redox potential impact on the sensing effect, a moderate negative correlation was observed between the standard reduction potential of metal ions and the failure ratio of CQD detection (r ≈ −0.5). Ions with strongly negative potentials (e.g., Mg^2+^, Ca^2+^, Na^+^, K^+^) were almost universally non-responsive, whereas ions with more positive potentials (e.g., Cu^2+^, Hg^2+^, Fe^3+^) showed significantly higher detection frequency.

This observation suggests that redox activity contributes to fluorescence modulation mechanisms in CQDs, supporting the hypothesis that electron transfer processes, rather than Lewis acid–base coordination alone, play a dominant role in many sensing systems.

**Figure 5:**
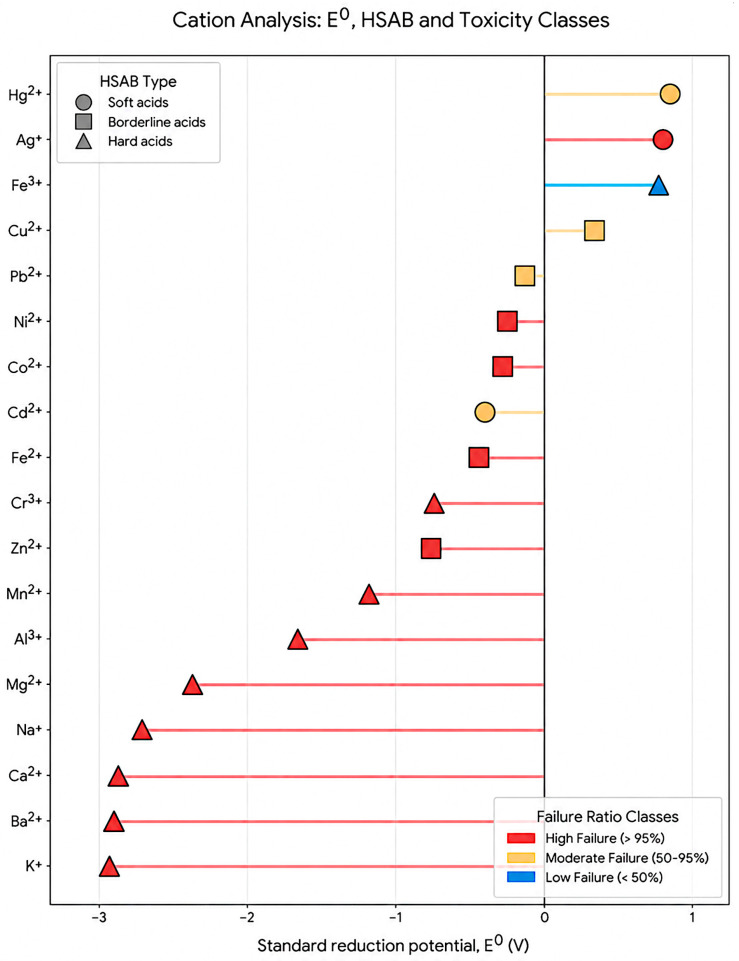
Two-dimensional mechanistic plot of CQD ion detection.

The statistical landscape presented in Figure 5 was derived directly from the comprehensive experimental data aggregated in Table 1. The dataset for this meta-analysis was compiled through a systematic literature search across the Scopus, Web of Science, and Google Scholar databases, covering the period from 2019 to early 2026. The search strategy employed keywords such as “carbon quantum dots”, “fluorescence ion sensing”, and “interference studies”.

The inclusion criteria required studies to report explicit selectivity data, specifically identifying both the target analytes and the list of ions that showed no significant response (reported as “non-interfering”). Studies with ambiguous or incomplete interference tables were excluded to maintain the integrity of the statistical work. A total of 60 independent research articles (Refs. [132,134,141,142,143,144,145,146,147,148,149,150,151,152,153,154,155,156,157,158,159,160,161,162,163,164,165,166,167,168,169,170,171,172,173,174,175,176,177,178,179,180,181,182,183,184,185,186,187,188,189,190,191,192,193,194,195,196,197,198] in Table 1) contributed to the final calculation of the failure ratio.

To ensure that claims regarding redox-inert ions—such as Zn (II), Mg (II), and Ca (II)—are statistically robust and not anecdotal, we established a minimum threshold of 10 explicit challenges (Tested\_total > 10) for an ion to be included in the visualization. This methodology confirms that the high failure rates for these cations are a reproducible result of their chemical nature across a vast diversity of CQD-based sensing systems.

This figure presents a two-dimensional plot combining HSAB types, standard reduction potential, and the observed CQD detection failure ratio. Each point represents one ion extracted from the compiled sensing dataset. The plot illustrates that successful CQD sensing is not governed solely by HSAB matching but emerges from the interplay between surface donor chemistry and ion electronic properties such as redox activity and heavy-atom effects.

### 5.2. Small Molecules and Reactive Species

For biological diagnostics and environmental monitoring, anion detection is essential. Fluoride (F^−^), cyanide (CN^−^), phosphate (PO_4_^3−^), nitrate (NO_3_^−^), sulfide (S^2−^), and halides have all been effectively sensed by CQDs. Mun Ryul Choi et al. [199] used quaternary ammonium functional groups on the surface of CQDs to achieve selective detection of perrhenate anions, even in the presence of excess chloride ions. This selectivity is attributed to photoinduced electron transfer, which is caused by electrostatic interactions between the cationic surface functions of CQDs and perrhenate ions. The system reliably sensed perrhenate concentrations up to 1 mM, with limits of detection of 87 and 208 μM. Mutendu C. Kungwa et al. [200] describe a method for creating N, S-CQDs complexed with lead ions as a fluorescent nanoprobe for fluoride ion detection. The precursors for sulphur, nitrogen, and carbon are sodium sulfide, glutamine, and citric acid, respectively. With a LOD of 78.03 nM, the nanoprobe complex demonstrated both sensitivity and selectivity towards fluoride ions when other ions were present. J.J.P. Oliveira et al. [201] showed the production of CQDs with a high fluorescent quantum yield (90.7%) using ethylene diamine and citric acid. These nanoparticles were used in a turn-on/turn-off sensor that uses iron ions to detect cyanide ions. At a concentration of 1.0 μg mL^−1^, the turnoff/turn-on sensor demonstrated a sensitivity and efficacy of 3.77 ppm for cyanide ion detection. Ömer Kaan Koç et al. [202] created a N-CQD-based fluorometric probe with a high quantum yield (73.10%) for the specific identification of nitro-explosive 2,4,6-trinitrotoluene (TNT) using ethylenediamine (EDA) and diaminocyclohexane (DACH) as nitrogen precursors and citric acid (CA) as a carbon precursor. In aqueous media, the LOD was 30.0 nM.

Oxidative stress, inflammation, and cellular signaling are all significantly impacted by reactive species such as hydrogen peroxide (H_2_O_2_), hydroxyl radicals (•OH), superoxide (O_2_•^−^), nitric oxide (NO), and peroxynitrite (ONOO). In biological and aquatic contexts, CQDs provide sensitive, real-time monitoring. Chao-Sheng Chen et al. [203] mixed lactose as the carbon source and tris(hydroxylmethyl)aminomethane (Tris) as the surface passivation reagent to prepare CQDs by the hydrothermal method. The CQDs’ remarkable sensitivity to the hydroxyl radical has led to their adoption as probes for fluorescence turn-off detection. When hydrogen peroxide is used as the source for ROS generation, the LOD can be as low as 0.1 μM under ideal circumstances. To detect nitric oxide, Marita A. Cardoso et al. employed the thermal breakdown approach to create CQD-based polymers such as folic acid (a biomolecule associated with the reactive oxygen and nitrogen (ROS/RNS) scavenging process), which quenched the CQDs’ fluorescence intensity by 25% [204].

### 5.3. Biological and Biomedical Targets

CQDs have been used extensively to detect bioactive chemicals that are relevant to biology and the environment, including hazardous organic compounds, glucose, dopamine, urea, and antibiotics. Clinical diagnostics, food safety, environmental monitoring, and industrial process control all depend on the sensitive identification of these species. When utilizing functional CQDs to assess the visibility and distribution of the analyte, fluorescence images of these molecules in living cells or tissues are preferred. To comprehend and track the emergence of antibiotic-resistant bacteria that are common in the area, it can be important to keep an eye on excessive antibiotic usage in humans, animals, and the environment [205]. Qian et al. [206] have reported the use of CQDs for sensing TC and quinolones. IFE significantly reduces the fluorescence of near-infrared emissive CQDs when these antibiotics are present. With a LOD of 6.3 nM for norfloxacin and 0.5 nM for oxytetracycline, the measurement of antibiotics is made possible by the quenching of the CQDs’ fluorescence. Wang et al. [207] used a one-pot hydrothermal method to prepare highly fluorescent CQDs. Up to 20.6%, its fluorescence quantum yield was high. The produced CQDs demonstrated linearity for OTC in lake water and urine, with a detection range of 0–50 µM and a LOD as low as 0.05 μM. Aysenur Aygun et al. [208] prepared Cu@N-CQDs via the hydrothermal technique. With a linear range of 0–140 μM and a LOD of 29.85 μM, the sensor demonstrated good sensitivity and selectivity for glucose. Aida Mousavi et al. [209] prepared a MIL-101-CQDs hybrid fluorescent probe sensor via a post-synthetic modification method for the detection of 6-mercaptopurine (6-MP) and demonstrated high selectivity and sensitivity for 6-MP. The fluorescence signal at 599 nm is quenched when 6-MP is added to this probe. For a concentration range of 1.09–6.5 nM, the limits of quantitation and detection were 1.32 nM and 0.37 nM, respectively.

CQDs have the potential to be the next generation of fluorescent probes for both in vivo and in vitro imaging. Based on the structure, surface functionalization, and type of cells, biocompatible CQDs may easily enter a variety of cells using endocytosis, a cell-penetration mechanism akin to macropinocytosis. This allows them to comprehend interactions with different proteins and monitor changes using bioimaging [210]. Pioneering work by Sun et al. [211] explored the use of CQDs for in vivo imaging in mice. According to their research, CQDs can be administered intravenously, intramuscularly, or subcutaneously. Each of these delivery modalities has a unique clearance profile, providing information about how the administration route affects the behavior and possible applications of CQDs in live imaging [212].

Yang et al. [213] developed deep red-emissive CQDs with a high quantum yield of 59%, demonstrating their effectiveness in both one-photon and two-photon bioimaging. These CQDs quickly circulated throughout the body, first accumulating in the liver, lungs, and kidneys before being effectively removed via the renal and hepatobiliary systems, according to biodistribution studies conducted in nude mice. Furthermore, CQDs’ capacity to interact with DNA makes them useful for forensic genetic investigation. CQDs improve forensic identification and genetic marker analysis by enabling highly sensitive and quick DNA detection through electrostatic and hydrogen bonding [214].

## 6. Selectivity in CQD-Based Sensors

The correlation between the heightened sensitivity and frequently diminished selectivity of CQDs in sensing applications is attributed to their distinctive structural characteristics. Their elevated chemical reactivity and numerous surface functional groups facilitate the detection of trace analytes (high sensitivity), yet simultaneously result in non-specific interactions with various coexisting species (low selectivity), thereby impeding their utility in the analysis of complex samples [215]. Within a single sensing platform, CQDs often show responsiveness toward several analytes. Their abundant redox activity, varied emissive states, and heterogeneous surface chemistry are the main causes of this multi-analyte behavior. Although this characteristic increases adaptability, it also brings up significant issues with analytical dependability and selectivity [117,216]. As a result, it is essential to increase selectivity by offering multiple tactics.

CQDs are useful for detecting objects like narcotics, explosives, and toxins because they can connect with a variety of molecules, including organic chemicals, metal ions, and biological molecules, due to functional groups like –OH, –COOH, and –NH_2_ [217]. When combined with fluorescence modulation, this selective interaction enables rapid detection even at trace levels [92]. Short-chain ligands can be used to functionalize the surfaces of these nanocrystals, increasing selectivity. Liquid ionic (IL)-based CQDs made under microwave radiation showed definite selectivity for different inorganic ions when just the ionic liquid anions were changed. Using 1-butyl-3-methylimidazolium bromide ([BMIM][Br]) and 1-butyl-3-methylimidazolium tetrafluoroborate ([BMIM][BF4]), the authors created N-doped CQDs with different surface compositions when exposed to microwave radiation [218]. Because of their unique radius and ionic conductivity, the anions Br− and [BF4]^−^ change the ionic liquid polarity, which in turn changes all ionic-microwave interactions. As a result, the resulting nitrogen-doped CQDs had a high surface concentration of imidazole groups ([BMIM][Br]), while the ([BMIM][BF4]) dots contained hydroxyl. These surface properties produced good selectivity over other metal ions and favored interactions with Cu^2+^ (by N-imidazole chelation) or Fe^3+^ (by high binding hydroxyl affinity) [218].

Moreover, the selectivity of CQDs can also be improved by using molecular recognition elements. Additionally, CQDs interact with proteins via hydrogen bonds and electrostatic interactions, thereby increasing their applications in biomolecular sensing. Protein identification in forensic situations, such as biomarkers or bodily fluids at crime scenes, depends on these interactions [214,219]. Putri et al. [220] developed a highly sensitive fluorescence sensor for *Escherichia coli* bacteria detection using a dual strategy. This method involved functionalizing CQDs with the antibiotic amikacin after co-doping them with N and S atoms (N, S-CQDs). Using citric acid as the carbon source, thiourea as the nitrogen- and sulfur-dopant, and varying concentrations of amikacin as the targeted moiety, amikacin-conjugated N, S-CQDs were prepared via microwave-assisted synthesis. Radiative relaxation pathways were enhanced, and new excitation energy traps were formed by doping with N and S, which supply additional electrons for n-type doping. The intensity of the fluorescence increased as a result. Furthermore, amikacin greatly increased the N, S-CQDs’ selectivity and affinity for *Escherichia coli*.

By utilizing two or more emission signals to compute a ratio, ratiometric and differential sensing using CQDs are sophisticated analytical techniques that improve detection accuracy by removing errors brought on by environmental factors, instrument fluctuations, or variations in sensor concentration. These techniques offer greater selectivity and sensitivity than conventional “turn-off” (quenching) or “turn-on” (enhancement) sensors, which depend on a single, absolute intensity measurement [221,222]. Using the distinct, cross-reactive, and multi-wavelength emission characteristics of various CQDs, differential sensing—which frequently makes use of sensor arrays—creates a “fingerprint” or pattern that can be used to distinguish between similar analytes. Identification is accomplished through machine learning and pattern recognition algorithms [223,224,225].

The same variability that permits wide analyte interaction can jeopardize repeatability, selectivity, and commercialization potential in the absence of controlled synthesis and thorough mechanistic research. To fully realize the potential of CQDs in analytical applications, future research must prioritize structural clarity and consistent characterization techniques.

## 7. Analytical Performance: LOD, LOQ, Response Time, and Signal Reliability

### 7.1. Definitions and Reporting Practices

To gauge the sensitivity of target analytes, LOD and LOQ of CQDs were developed. The smallest amount of a material that can be reliably identified from a blank and detected with a fair degree of certainty is known as the limit of detection, or LOD. However, the lowest analyte concentration that can be accurately measured is known as the limit of quantitation (LOQ). Depending on the analytical tool, a substance’s mass or concentration can be used to determine its quantity [226].

Assessing analytical performance via LOD, LOQ, and signal dependability presents many prevalent problems that may undermine technique validation. Fluctuations in background noise or non-linear calibration curves sometimes result in erroneous LOD/LOQ calculations, which may either overestimate or underestimate actual detection limits. Matrix effects in intricate samples impede signal distinction from interferences, especially in methodologies such as HPLC or GC-MS, requiring substantial statistical repetitions and matrix-matched standards. Furthermore, inconsistencies between regulatory standards and variable operational variables, such as temperature or sample stability, further compromise signal dependability, necessitating sophisticated corrective procedures for consistent results [227,228]

### 7.2. Comparison of LOD and LOQ Across CQD-Based Sensors

The transduction technique and the target analyte’s interaction with the dot’s surface determine LOD and LOQ in CQD-based sensing. Because of its ease of use, fluorescence is the most widely used technique; nevertheless, electrochemical techniques frequently offer higher sensitivity for particular metal ions and biomolecules [229,230].

Experimental circumstances and sample matrix effects have a significant impact on the analytical performance of CQD-based sensors, especially LOD and LOQ. The signal-to-noise ratio and apparent detection limits can be affected by factors like pH, ionic strength, and background constituents. For example, fluorescence quenching behavior in quantum dot-based sensors and related systems has been demonstrated to be impacted by pH and ionic strength, suggesting that changes in solution chemistry can either suppress or enhance signal outputs in real matrices when compared to ideal buffers [231,232]. While electrochemical measurements may be hampered by electrode fouling and non-specific adsorption, which increases baseline noise and decreases sensitivity, background scattering and competing interactions can further shift LOD values in fluorescence detection in complex samples containing natural organic matter or other interferents [233,234]. Furthermore, discrepancies in the computation of LOD and LOQ can make cross-study comparisons more difficult and highlight the necessity of uniform reporting to precisely evaluate performance under actual circumstances [235].

### 7.3. Response Time

Response time is a critical metric for assessing the efficacy of fluorescence-based nanosensors. It denotes the duration necessary for a sensing device to attain a stable fluorescence signal following the introduction of the target analyte. In practical applications like environmental monitoring and biological diagnostics, a rapid response time is essential since it facilitates prompt detection and real-time surveillance of harmful metal ions. Therefore, response time is considered one of the most important analytical parameters in sensor evaluation, following the LOD and LOQ.

In Classical spectrometric methods, Marczenko define analytical performance at the macroscale by the equilibrium time required for phase distribution and complex formation before optical measurement; therefore, the principle of the time required for analyte–ligand interaction is essential across analytical systems [236,237]. In CQD-based sensors, this interaction occurs at the nanoscale through surface functional groups, where binding events and consequent electron or energy transfer processes influence the optical response time. Consequently, a response time in these systems can be viewed as a kinetic alternative to the equilibration time, where the interfacial reaction rate governs signal formation.

CQD-based fluorescent sensors demonstrate response times ranging from a few seconds to several minutes, depending on the sensing mechanism and experimental conditions. For example, CQDs-modified graphene transistor sensors for Cu^2+^ detection have shown response times on the order of seconds due to strong coordination interactions between CQDs and copper ions [236]. Similarly, fluorescence-based CQD sensors for heavy metal ions often reach signal stabilization within a few minutes after the addition of the analyte [238]. In some systems, fluorescence quenching processes can reach equilibrium within approximately 5 min, indicating relatively fast sensing kinetics for Fe^3+^ detection using CQDs [239].

While the LOD is frequently employed to assess the sensitivity of nanosensors, response time is equally crucial in establishing their practical applicability, as the correlation between response time and detection sensitivity is closely linked to the interaction kinetics between CQDs and analyte. Faster binding kinetics generally lead to more rapid fluorescence modulation, which improves detection speed without compromising sensitivity. Surface functionalization of CQDs with heteroatoms or specific ligands can simultaneously enhance binding affinity and accelerate electron transfer processes, leading to both lower detection limits and shorter response times. Consequently, the rational design of CQDs’ surface chemistry plays a critical role in optimizing both parameters simultaneously. For instance, Gonzalez et al. demonstrated biomass-derived CQDs from jojoba leaves for Fe^3+^ detection with an ultralow LOD of 0.018 μM and a rapid response time of ~3 min, illustrating the potential of green-synthesized CQDs for ultrasensitive and fast sensing applications [134]. In contrast, Zherui Wan et al. reported N-doped CQDs as an “on–off” fluorescent probe for Fe^3+^ ions with a relatively higher LOD of 0.510 μM. As a result, the response time was about 7 min, highlighting the relation between LOD and response time [240]. Hasan Shabbir et al. prepared CQDs for Fe^3+^ detection with a LOD of 0.49 ppm. They have proved that the detection efficiency of CQDs was increased up to 200% after 30 min., thus it is recommended to use this length of time to optimize detection [133].

### 7.4. Signal Stability, Reproducibility, and Real-World Constraints

Despite their remarkable photostability, CQDs can nevertheless suffer from photobleaching and signal drift under some situations, which can impair their capacity to sense and image [25,241]. The irreversible loss of fluorescence brought on by photon-induced chemical damage or change is known as photobleaching. Surface-adsorbed chromophores can be transformed into non-emissive forms by UV or high-intensity light, frequently through isomerization, before these optically active centers are destroyed at higher fluences [242,243].

The term “signal drift” describes how the recorded fluorescence signal gradually shifts or becomes unstable over time, which can result in measurement inaccuracies. It is caused by environmental sensitivity, such as pH, temperature, or interfering ion fluctuations, surface fouling, or instrumental factors [244,245,246].

When the same synthesis procedure is repeated, there can be notable variations in the physical and optical characteristics of CQDs, including size, surface chemistry, and quantum yield. This is known as batch-to-batch variability. A significant obstacle to industrial-scale and biomedical applications is this inconsistency [247,248,249]. Because it is more difficult to maintain precise control over nucleation and growth in larger reactors, translating lab-scale synthesis to industrial volumes frequently degrades reproducibility. It is caused by precursor inconsistencies (biomass sources and heterogeneity), synthesis conditions, and scale-up challenges [250,251,252]. Techniques for lowering variability include microwave-assisted synthesis, post-synthesis purification, continuous manufacturing, and standardized precursors [251,253].

## 8. Electrochemical and Electrochemiluminescent CQD-Based Sensors

### 8.1. Electrochemical Sensors

Electrochemical sensors surpass fluorescent, colorimetric, and immuno-like sensors owing to their enhanced sensitivity, expedited reaction times, and capacity to identify low analyte concentrations. They provide real-time surveillance, are economical, and can be downsized for portability. Moreover, they operate throughout intricate matrices without necessitating substantial sample preparation [254]. CQDs are widely used in the creation of electrochemical sensors. They work as nanocarriers for bioreceptors, electroactive labeling components, nanoenzymes, electron transfer promoters, and nanocatalysts [255]. CQDs are employed as transducer elements in sensing technology, either alone or in conjunction with other substances. They are perfect for creating extremely effective electrochemical sensing platforms because of their special blend of electrical and optical properties [256]. For electrochemical sensing applications, these QDs’ precise sizes and electrical characteristics are essential. These sensors have very low detection limits in addition to excellent sensitivity brought on by strong electron mobility, improved selectivity, and quick reaction times. They work well for extended use and are stable in a variety of environmental circumstances [257]. Glassy carbon, gold, or carbon paste electrodes are examples of electrode materials on which CQDs are immobilized. To improve the sensor’s sensitivity and selectivity, the CQDs can be functionalized with particular molecules or directly placed onto the electrode surface. By means of electrochemical reactions taking place at the electrode surface, these sensors are able to identify analytes. When target analytes are present, the CQD-modified electrode’s conductivity or electron transfer kinetics change, producing detectable electrochemical signals [235]. Zahra Nazari et al. [258] synthesized a novel electrochemical sensor based on a carbon paste electrode (CPE) modified with CQDs for detecting dopamine (DA) with uric acid and ascorbic acid. The electrode produced satisfactory results with a concentration range of 0.1–50 μM in phosphate buffer and a LOD of 0.046 μM when it was successfully utilized to determine DA in real samples. Jillian Gamboa et al. [229] developed a CQDs electrochemical sensor to enable selective detection of dopamine with a LOD of 55 nM and a broader detection range (1–500 µM).

Because of their superior electron transfer capability, high conductivity, and surface passivation, CQDs can also demonstrate a potent synergy between their photochemical and electrochemical activities. By reducing charge carrier recombination and increasing light absorption, this synergy enables CQDs to function as electron mediators, sensitizers, and active sites in photoelectrochemical (PEC) systems, frequently greatly improving the performance of photoanodes [118,259]. Qiang Zhao et al. [260] assembled CQDs and Au nanoparticles (NPs) on the surface of a three-dimensional (3D) spherical Bi_2_MoO_6_ (BMO) nanostructure with surface oxygen vacancies (SOVs), which exhibited an effective strategy to enhance the utilization of up-conversion emission and plasmonic energy. Lei Ding et al. [259] synthesized Bismuth vanadate co-doped with Mo and W surface with CQDs and Fe MOF nanoparticles. The incorporation of CQDs resulted in a significant increase in the photocurrent density while enhancing its light absorption ability. This improvement is due to the introduction of defects brought by CQDs.

### 8.2. Electrochemiluminescence (ECL)

Electrochemiluminescence (ECL) is an effective and efficient method for sensing applications since the pioneering studies of Hercules and Bard in the 1960s [261,262].

By combining the unique benefits of electrochemistry and spectroscopy, ECL produces excellent sensitivity, low synthesis costs, high stability, functional flexibility, low background noise, good catalytic activity, perfect opto-electronic properties, and quick reaction times [263]. Most ECL processes consist of four steps: light emission, homogeneous chemical reactions, excited state species generation, and redox reactions at electrodes [264]. Since 2002, when Bard initially noticed the ECL phenomenon of Si QDs [18], ECL studies of QDs with various structures and compositions have been studied [265,266]. It is theoretically possible to quantitatively identify any chemicals that can alter the final ECL emission by interfering with (inhibiting or promoting) the aforementioned stages [264].

Functional groups or π–π interaction at several edge sites allow analytes to interact with the surface of QDs, which is a crucial parameter for sensing applications. Furthermore, depending on the intended use, sp^2^ hybrid orbitals can regulate charge transfer in functionalized QDs. Additionally, these QDs exhibit electrochemiluminescence (ECL) characteristics, which allow an electric potential to stimulate them. These states release light that can be measured for analyte detection after de-excitation takes place [267,268]. Because of their tunable emission, excellent stability, and inexpensive manufacturing, CQDs are useful electrochemiluminescence (ECL) emitters. They generate light via co-reactant routes or radical annihilation, frequently competing with conventional emitters like Ru(bpy)_3_^2+^ [269]. Light-emitting excited states are produced when CQD radicals engage with coreactants such as K_2_S_2_O_4_. Compared to Ru standards, CQD^−^ donates electrons to sulfate radicals with up to 96% relative efficiency [269].

Due to reduced backgrounds, no need for an external light source, and electrochemical control, CQD-based ECL sensing typically performs better than fluorescence-based techniques in detection limits and signal-to-noise ratios. For example, meta-analyses of quantum dot sensors reveal a geometric mean LOD of 38 nM for fluorescence-based techniques and 0.109 pM for chemiluminescent/ECL-based ones, a difference of almost five orders of magnitude. Additionally, because the light emission is controlled both spatially and temporally at the electrode surface, ECL offers better signal-to-noise ratios and a wider dynamic linear range. ECL is the preferred modality for ultra-sensitive clinical diagnostics and the detection of trace environmental toxins, whereas fluorescence is still preferred for multiplexing and ease of high-throughput operation [263,269,270,271,272,273].

Because of their unique characteristics, CQDs are frequently altered as electrode surface substrates for organic molecules, biomolecules (enzymes, antibodies, DNA), and nanoparticles [264]. H Ji et al. [274] synthesized N-doped CQDs modified with enzymes as the substrate, which promoted the electron transfer between the enzyme and electrode and improved the electrocatalytic activity for glucose detection. Furthermore, it can be used for metal ion sensing. Zhang et al. [275] observed a dual-peak ECL behavior of CQDs in tetrabutyl ammonium bromide (TBAB) ethanol solution and applied it in iron ion detection. They found that the electron injection into the conduction band of CQDs led to the first ECL peak, and ion annihilation reactions produced another ECL peak. The variations between CQDs sensor types are illustrated in Table 2.

## 9. Hybrid and Multimodal CQDs-Based Sensing Platforms

Hybrid and multimodal sensing platforms utilizing CQDs amalgamate the inherent photoluminescence of CQDs with other nanomaterials to attain improved sensitivity, selectivity, and dual-signal detection. These platforms usually combine the structural, magnetic, or electrochemical benefits of complementary materials with optical sensors [272,276].

Surface modification not only improves the characteristics but also creates new reaction sites that allow for more surface reactions. CQDs’ surfaces have been modified using calcium, magnesium, scandium, manganese, iron, copper, nickel, cobalt, zinc, gallium, bismuth, molybdenum, silver, gold, gadolinium, etc. [277,278,279]. Doping creates HOMO-LUMO energy band gaps, which enhance the physicochemical characteristics of CQDs. The intrinsic structure of CQDs varies as a result, and eventually the electronic distribution shifts due to the creation of n-type or p-type carriers [280]. Quan Xu et al. [281] fabricated zinc-doped CQDs with a quantum yield of 35%. The zinc ion charges serve as a surface passivating agent and hinder the aggregation of graphene π–π stacking, leading to an increase in the QY of the Zn-CQDs, which were applied for the ultra-trace detection of Hg^2+^ with a LOD of 0.1 μM. Chunmei Li et al. [282] prepared Cr-CQDs by hydrothermal pathway using tris(2,4-pentanedionato) chromium (III) and polyethyleneimine as precursors. The Cr-CQDs have a graphene-analogous structure and display blue-green fluorescence with excitation/emission maxima at 350/466 nm, a 20% quantum yield, and excitation-independent emissions. The Cr-CQDs were used as a fluorescence probe for *p*-nitrophenol with a linear working range that extends from 0.8~150 μM and a 0.27 μM lower detection limit.

Because of their ductility, low specific gravity, intriguing physical characteristics, and ease of processing, polymer matrices are special. However, their modulus is far lower than that of metals and ceramics. Consequently, because of the reinforcing nature of QDs in polymer matrices, their integration is extremely relevant [283,284]. Fluorescent CQDs are incorporated into polymer matrices to develop flexible, biocompatible devices for the detection of pollutants, ions, and biomarkers. These composites take advantage of the low toxicity, stability, and adjustable fluorescence of CQDs for uses such as environmental sensing and wearable health monitors [285]. Dinh Khoi Dang et al. [286] prepared CQDs that were incorporated into a polymeric matrix composed of 3-aminopropyltriethoxysilane and tetraethyl orthosilicate in the presence of glucose, followed by a molecular imprinting process. The binding holes in the resultant polymeric particles matched the spatial layout of glucose’s hydroxyl groups. In the intracellular fluid-mimicking solution, the imprinted polymer demonstrated successful glucose identification, reaching a LOD of 29.4 nM with glucose concentrations ranging from 25 nM to 25 mM. This luminous sensor shows great promise for practical uses in intracellular glucose monitoring. Neha Garg et al. [287] synthesized highly fluorescent hybrid CQDs with poly-N isopropylacrylamide for Tryptophan (Trp) sensing with high accuracy and selectivity, with a wide linear range of 10–500 μM, and a LOD as low as 0.1 μM.

In order to obtain greater sensitivity, selectivity, and spatial resolution, multimodal sensing combines optical and electrochemical modalities into a single platform, integrating two different detection concepts. The non-invasiveness, speed, and sensitivity of optical techniques, including colorimetry, fluorescence, surface plasmon resonance, and surface-enhanced Raman spectroscopy, are highly regarded. These techniques, when coupled with the accuracy and precision of electrochemical transducers, produce dual-readout platforms that provide qualitative and quantitative insights, increasing detection ranges and enhancing dependability, especially at low concentrations [288,289]. The development of this field has been greatly aided by materials with both colorimetric and electrochemical characteristics. Zhou et al. [290] demonstrate the effectiveness of an EC-PEC sensor for Cr(VI) detection. In order to improve electrochemical performance, the researchers combined nitrogen-doped carbon with bismuth oxy iodide (BiOI), a potent photosensitizer, to create a porous composite. Efficient photoelectron separation and electron transfer are made possible by the BiOI/N–C heterojunction. By amplifying both EC and PEC signals, this synergy makes it easier to photo-reduce Cr (VI) and achieve ultralow detection limits.

## 10. Future Perspectives and Design Principles

CQDs exhibit inherent photochemical activity and broad-spectrum sensitivity, enabling the detection of metal ions, biomolecules, and pH variations primarily through surface-mediated interactions. This feature provides exceptional sensitivity, often achieving LODs in the nanomolar to picomolar range; however, it also results in intrinsic cross-sensitivity and limited selectivity due to nonspecific adsorption and quenching mechanisms.

Advancements in CQD sensors necessitate a shift from solely optimizing sensitivity to adopting a design philosophy centered on enhancing selectivity. Previous efforts should shift the focus from minimizing isolated LOD in idealized media to targeted surface passivation guided by coordination chemistry, redox potential, and band-structure engineering. For instance, analyte-specific heteroatom doping, such as nitrogen to improve Lewis base properties, and sulfur or phosphorus for soft-metal affinity, and bifunctional ligands for mercury ion chelation, can be systematically selected through density functional theory (DFT) simulations based on binding energies and frontier orbital overlaps. Hybridization with biomimetic elements, such as aptamers, metal–organic frameworks (MOFs), or molecularly imprinted polymers (MIPs), can distinguish target-specific responses from non-specific binding in applications that demand high discrimination.

Transitioning from phenomenological explanations, such as inner filter effects, static/dynamic quenching, or photoinduced electron transfer (PET), to quantitative verification is critical for mechanistic understanding. To enhance inter-study repeatability and mechanistic comparability, future research should focus on techniques such as time-resolved fluorescence spectroscopy, spectrum overlap integrals for inner filtering, and nonlinear Stern–Volmer analysis to elucidate quenching mechanisms.

The electrochemical (EC) and electrochemiluminescence (ECL) properties of CQDs offer complementary transduction channels to fluorescence. They leverage co-reactant compatibility and tunable redox potentials. While ECL enhances signals through electron-transfer cascades, resulting in light emission, achieving LODs below 1 nM with over a hundred-fold signal amplification compared to steady-state voltammetry. EC sensing effectively detects redox-active analytes via electrocatalytic currents. Nonetheless, challenges persist, including limited understanding of ECL processes beyond annihilation or co-reactant pathways and electrode passivation caused by CQD aggregation, which compromises reproducibility.

Future developments should prioritize conductive composites, such as CQD-graphene hybrids for antifouling surfaces, and co-reactant engineering. Integrating EC/ECL with fluorescence in multimodal arrays could facilitate comprehensive analyte profiling, with machine learning algorithms interpreting spatiotemporal signal patterns to achieve high specificity because it can extract selectivity from multivariate fluorescence data, achieving classification accuracies exceeding 95% in complex mixtures.

The translational success of CQD sensors hinges on rigorous benchmarking in real-world conditions: photostability, reversibility, scalability through continuous-flow synthesis, and validation of key performance parameters, including LOD, selectivity, and response time, in authentic matrices, under standardized interference conditions with surplus competitors.

Fundamentally, the future of CQD sensing lies in harnessing its extensive reactivity rather than suppressing it. This entails comprehensive validation across optical, electrochemical, and ECL domains, array-enabled pattern recognition, mechanistic verification, and the application of computational design to optimize their multifaceted capabilities.

## 11. Conclusions

CQDs emerge as highly versatile sensing nanomaterials, utilizing their inherent photophysical, electrochemical, and electrochemiluminescent properties for detecting pH, metal ions, biomolecules, and environmental analytes. Their broad responsiveness through surface-mediated interactions results in ultralow LOD. However, beyond empirical optimization, it is necessary to focus on selectivity engineering.

This study diverges from traditional literature reviews that concentrate solely on successful detections by introducing a negative chemical space analysis, which includes non-detected ions from each report. This approach significantly enlarges the dataset and facilitates data-driven identification of the critical physicochemical factors influencing CQD sensing. The findings indicate that CQD sensing efficacy is not exclusively governed by HSAB matching, but is also significantly affected by redox activity, heavy-atom effects, and surface donor chemistry.

This review also delineates a clear progression from single-modality EC/ECL sensors and sensitivity-driven fluorescence “turn-off/on” techniques to systematically designed, multimodal platforms. Significant advancements involve integrating cross-reactive arrays, analyzed by chemometrics and computational surface engineering, with validated quenching/redox mechanisms, transforming intrinsic cross-sensitivity into multivariate selectivity with over 95% accuracy. For practical applications, the standardization of CQD synthesis and the comprehensive validation in real matrices remain crucial. Ultimately, the future of CQDs lies in exploiting their polychromatic reactivity as the foundation for next-generation sensing systems aimed at clinical diagnostics, environmental monitoring, and other fields, rather than merely emulating molecular probes.

## Figures and Tables

**Table 2:** Comparative evaluation of LOD, LOQ, mechanisms, advantages, and limitations of CQD-based sensing platforms.

CQDs Sensor Type	LOD Range	LOQ Range	Mechanism	Advantages	Challenges
Fluorescence	20 nM–1.5 µM	65 nM–5.0 µM	Quenching (Turn-off) or Recovery (Turn-on)	High sensitivity, rapid response	Non-specific quenching, prone to matrix interference.
Electrochemical	0.04 nM–100 nM	0.13 nM–330 nM	Redox reactions, enhanced conductivity	Exceptional sensitivity for metal ions, simultaneous detection	Complex electrode modification
pH Sensors	0.01–0.5 pH units	0.03–1.5 pH units	Protonation/Deprotonation of surface groups	Linear response over broad pH ranges	Highly sensitive to the ionic strength of the matrix
Metal-Ion	1.0 nM–0.5 µM	3.3 nM–1.6 µM	Chelation or coordination with surface ligands	Specificity through functionalization	Competing ions often cause false positives.

## Data Availability

No new data were created or analyzed in this study. Data sharing is not applicable to this article.
